# Encoding of 2D Self-Centered Plans and World-Centered Positions in the Rat Frontal Orienting Field

**DOI:** 10.1523/JNEUROSCI.0018-24.2024

**Published:** 2024-08-12

**Authors:** Liujunli Li, Timo Flesch, Ce Ma, Jingjie Li, Yizhou Chen, Hung-Tu Chen, Jeffrey C. Erlich

**Affiliations:** ^1^New York University-East China Normal University Institute of Brain and Cognitive Science at New York University Shanghai 200062, Shanghai, China; ^2^New York University Shanghai, Shanghai 200124, China; ^3^Shanghai Key Laboratory of Brain Functional Genomics (Ministry of Education), East China Normal University, Shanghai 200062, China; ^4^Oxford University, Oxford OX1 2JD, United Kingdom; ^5^Sainsbury Wellcome Centre, University College London, London W1T 4JG, United Kingdom

**Keywords:** frontal cortex, motor planning, neural networks, neurophysiology, reference frame, rodent

## Abstract

The neural mechanisms of motor planning have been extensively studied in rodents. Preparatory activity in the frontal cortex predicts upcoming choice, but limitations of typical tasks have made it challenging to determine whether the spatial information is in a self-centered direction reference frame or a world-centered position reference frame. Here, we trained male rats to make delayed visually guided orienting movements to six different directions, with four different target positions for each direction, which allowed us to disentangle direction versus position tuning in neural activity. We recorded single unit activity from the rat frontal orienting field (FOF) in the secondary motor cortex, a region involved in planning orienting movements. Population analyses revealed that the FOF encodes two separate 2D maps of space. First, a 2D map of the planned and ongoing movement in a self-centered direction reference frame. Second, a 2D map of the animal’s current position on the port wall in a world-centered reference frame. Thus, preparatory activity in the FOF represents self-centered upcoming movement directions, but FOF neurons multiplex both self- and world-reference frame variables at the level of single neurons. Neural network model comparison supports the view that despite the presence of world-centered representations, the FOF receives the target information as self-centered input and generates self-centered planning signals.

## Significance Statement

Motor planning in the real world involves complex coordinate transformations: eye to head to body, hand, etc. Typical rodent tasks (e.g., go/no-go or two-alternative) are too simple for studying these processes. We trained rats to perform delayed, visually guided movements in multiple directions from varied start positions to explore coordinate systems in planning. We found that the frontal orienting field (FOF) encodes two separate maps: one for planning in self-centered coordinates and another for encoding current position in world-centered coordinates. Additionally, position and direction information are multiplexed at the single-neuron level. Our task and findings provide a foundation for understanding complex motor planning at a circuit level.

## Introduction

We use multiple reference frames to represent the world. For example, as you plan a movement to reach for your morning coffee, the arm region of motor cortex may represent the goal in an arm-centered reference frame and your frontal eye field (FEF) represents the goal in an eye-centered reference frame. Other areas of your brain may represent the cup relative to the room or table or the milk or sugar. During sensorimotor behaviors, sensory information is initially represented in sensor reference frames and motor commands are finally represented in muscle reference frames. Since the sensors and effectors are embodied, we can think of these representations as being in self-centered (or egocentric) reference frames, i.e., they are reference frames that move around with the subject as they move through the world. However, our lived experience is in a world-centered (or allocentric) reference frame: we feel as if we move around and make decisions in a stable world. Moreover, allocentric representations are found in a wide range of brain regions (O’Keefe and Nadel, 1978; Taube et al., 1990; Hafting et al., 2005; Wilber et al., 2014). Thus, a full understanding of the neurobiology of motor planning needs to address the question of where and how these reference frame transformations take place (Andersen and Mountcastle, 1983; Andersen et al., 1985, 1990; Cohen and Andersen, 2002).

The neural mechanisms of motor planning in rodents have been extensively studied in two-alternative forced choice (2AFC) and go-nogo tasks (Erlich et al., 2011; Sul et al., 2011; Guo et al., 2014; Chen et al., 2017). Converging evidence has implicated the frontal orienting field (FOF), a subregion of the secondary motor cortex (M2), as a cortical substrate for planning orienting movements (Erlich et al., 2011; Hanks et al., 2015; Olson et al., 2019), especially when those plans require flexible sensorimotor processes (Erlich et al., 2015; Siniscalchi et al., 2016; Bao et al., 2023). Quantitative models suggest that the FOF is a part of a bistable attractor network for short-term memory and decision-making in 2AFC orienting movement planning (Hanks et al., 2015; Kopec et al., 2015; Piet et al., 2017). Similar work in the mouse anterior lateral motor cortex during directional licking also identified discrete bistable attractor models as best accounting for observed neural activity and perturbation results (Li et al., 2016; Inagaki et al., 2019). Bistable attractor models are currently state of the art for 2AFC motor planning, but they are ambiguous as to the spatial reference frame of the neural coding. Do they represent the planned movement direction in an self-centered reference frame, the target position in an world-centered reference frame, or a “decision” in an abstract reference frame (Charlton and Goris, 2024)? Given the limitations of those behavioral paradigms, the answer is largely unknown.

The FOF is a potential site to integrate egocentric and allocentric spatial representations, as it receives input from the posterior parietal cortex (Reep et al., 1994) and the retrosplenial cortex (Yamawaki et al., 2016), both of which exhibit egocentric as well as allocentric spatial representations (Wang et al., 2020). To test the reference frame of action planning representation in the FOF, we designed a multi-directional, multi-positional orienting task that could distinguish between allocentric and egocentric reference frames. We found preparatory and movement-related activity in the FOF that were in the egocentric reference frame. Interestingly, allocentric target position information was also encoded in the FOF, but emerged later than direction encoding. The allocentric encoding represented the *current* rather than the upcoming position of the animals, indicating the allocentric activity did not play a primary role in action planning. These two reference frames were multiplexed at the single-neuron level: task-related activity was best described as an egocentric direction tuning multiplicatively modulated by the allocentric current position. These observations were best captured by a recurrent network model whose input and output were both in the egocentric reference frame, further supporting our conclusion that planning in the FOF takes place in an egocentric coordinate frame.

## Materials and Methods

### Subjects

Three adult male Sprague-Dawley rats and one adult male Brown Norway rat (Vital River, Beijing, China) were used in this study. For a portion of the experiments presented here, rats were placed on a controlled-water schedule and had access to free water 20 min each day in addition to the water they earned in the task. For some experiments, rats were given free access to a 4% citric acid solution (Reinagel, 2018), in addition to the normal water they earned in the task. They were kept on a reversed 12 h light-dark cycle and were trained during their dark cycle. Animal use procedures were approved by New York University Shanghai International Animal Care and Use Committee following both US and Chinese regulations.

### Experimental design

#### Behavioral training apparatus

**Figure 1.**
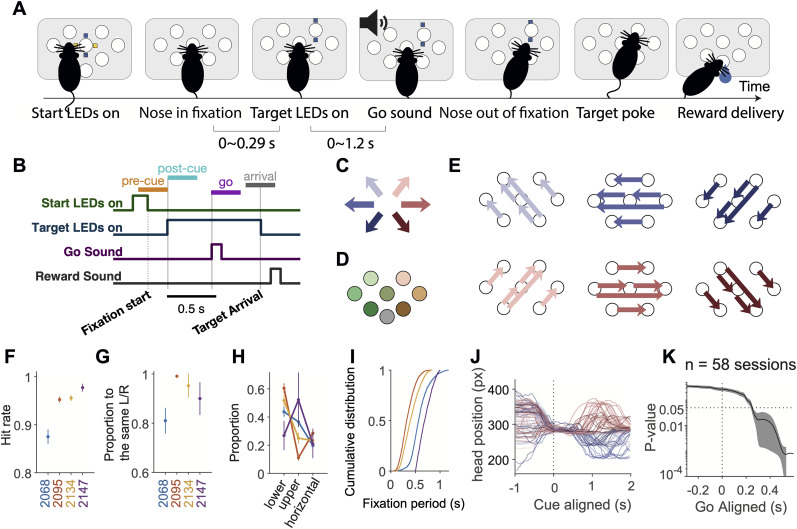
A visually guided multi-directional orienting task in rats. ***A***, Schematic of the task. Each trial began with the onset of a pair of blue and yellow LED, cuing the rat to nose poke into the start port. The start LED extinguished upon arrival in the start port. After a short delay, a blue LED illuminated, indicating the target port. After a go sound, the rat withdrew from the start port and poked into the target port. Water reward was delivered for correctly performed trials. ***B***, Timeline of a trial in a typical session. Bars above the timelines illustrate the time windows used in the subsequent analyses. “Pre-cue,” −300 to 0 ms from visual cue onset. “Post-cue,” 0–300 ms from visual cue onset. “Go,” 0–300 ms from go sound. “Arrival,” −150 to 150 ms from target poke. ***C***, The color scheme of the six movement directions. ***D***, The color scheme of the seven port positions. ***E***, The 30 movement trajectories consisted of 6 directions (shown in *C*). Each direction was associated with five possible trajectories, and there were four possible target ports associated with each direction. Each session only contained a subset of 16–24 trajectories among these trajectories, and these sessions were pooled together for analyses (see Methods for the details). ***F***, The fraction of correct trials among all the completed trials. Dots and error bars denote mean ± s.e. across sessions. ***G***, The proportion of errors made into the same left/right direction as instructed, among trials starting from the central port for each animal. Dots and error bars denote mean ± s.e. across sessions. ***H***, The proportion of errors whose movement directions were lower, upper, or horizontal compared to the instructed movement direction. For each subject, the three dots and error bars denote the mean ± s.e. across sessions for the fraction of lower, upper, and horizontal errors. For subject 2095 and 2134, there were significantly more downward errors than upward errors. ***I***, The range of fixation periods experienced by each rat, shown as a cumulative distribution. The color of the lines correspond to the colors of the labels in *F*. ***J***, Example traces of the horizontal head position in the video pixel space during trials starting from the central port. The traces were aligned to the visual cue onset. Each line is a trial, and the colors indicate the movement directions. ***K***, There was no significant correlation between the horizontal head position and the planned movement direction before the go cue. The line indicates *p*-value of the movement direction modulation on the horizontal head position across time aligned to the go sound. All the correctly performed trials were included and the effect of start position was captured in the random effect. Line and error bars, mean ± s.d. of the *p*-values over the 58 sessions.

Rats were trained in custom behavioral chambers, located inside sound- and light-attenuated boxes. Each chamber (23 × 23 × 23 cm) was fit with a vertical 2D port wall that had 7 operant ports and 1 reward delivery port, with speakers located on the left and right side (Fig. 1). Each operant port contained a pair of blue and a pair of yellow light emitting diodes (LED) as visual cues, as well as an infrared (IR) LEDs and photo-transistors for detecting rats’ interactions with the ports. The reward delivery port contained a stainless steel tube for delivering water rewards.

#### Behavioral task

The task timeline is described in detail in Results and Figure 1. In one rat (2147), in addition to the main type of timeline, which we denote as “target during fixation,” there were two other types of trial timelines: “target before fixation” and “target after go.” These types of timelines existed on different days from the main type of timeline and were not included in the analysis.

The duration of the fixation period was dynamically adjusted for each animal, ranging between 0 and 1.2 s (Fig. 1*I*). A trial was considered a fixation violation if the rat withdrew from the start port before the go sound. In fixation violation trials, an “error” sound was delivered and the trial was aborted.

Thirty movement trajectories were involved in the final behavioral stage (Fig. 1*E*).

In rat 2068, 2095, and 2134, there were two session types interleaved across days.

The “reference” sessions only involved the four shorter movements. Each of the 6 directions had 4 movement trajectories of the same distance (short trajectories in Fig. 1*E*), which yielded 24 movement trajectories in total. Seven hundred and seventy-one neurons were recorded from these sessions.

The “distance” sessions involved the two shorter movements and one long movement that crossed the central port for each direction (trajectories that crossed the central port in Fig. 1*E*), which yielded 3 movement trajectories for each direction and 18 movement trajectories in total. A total of 426 neurons were recorded from these sessions.

In rat 2147, there were 4 movement directions that were all oblique. Each direction had 4 trajectories of the shorter distance, summing up to a total of 16 trajectories. Twenty-seven neurons were recorded from these sessions.

Data from the “reference” sessions, “distance” sessions and the sessions from 2147 were pooled together in our analysis, as long as the session had at least 8 trials for each direction, start position and target position.

#### Video tracking

Videos were acquired at 30 frames per second with one Raspberry Pi Camera at the top of the rig. Fifty-eight sessions had videos acquired.

#### Behavior training pipeline

Rats went through a series of training stages, which had mainly two phases: (1) the operant conditioning phase, and then (2) the multi-directional orienting phase.

In the operant conditioning phase, rats became familiar with the training apparatus and learned to do a one step poke into the illuminated choice port. The first stage was to learn to collect reward from the reward delivery port. Each trial began with the illumination of the reward port, and water reward was immediately delivered upon port entry, followed by a short timeout period before the start of next trial. After the rats learned to poke into the reward port reliably (not missing any reward for six trials in a row), they proceeded to the next training stage. In the second stage, we turned on the LED for several random ports at the beginning of each trial. Rats had to first poke into any illuminated choice port before gaining water reward from the reward delivery port. The number of the illuminated port will gradually decrease to one after several trials when animals started to learn. After animals were able to poke the only illuminated choice port successfully for six trials in a row, we will upgrade them to the second training phase.

In the first stage of the multi-directional orienting phase, the start port was always the central port, and the target port was one of the six surrounding ports. Rats needed to poke into the start port to trigger the target port light, and then poke into the target port after a delay. Trials of the same movement trajectory were repeated until the animal could do several correct trials in a row. The training of “fixation” at the start port was introduced in this phase. Fixation means the rat had to keep its nose in the start port for a given time period (typically > 0.5 s). Fixation duration was initially 0.02 s early in the training, and was gradually increased based on an adaptive steps method: the fixation duration would increase on a successful fixation, and decrease when the fixation failed. We trained subjects to perform fixation for at least 0.6 s before the surgery, and fixation duration always jittered across trials in recording sessions. However, the speed to recover fixation after the surgery varied across subjects, thus we manually adjusted the fixation duration for each subject. In 2095, the mean fixation period in each session was shorter than 0.2 s for around 30% of sessions. In other subjects, the mean fixation period was typically longer than 0.4 s (Fig. 1*I*). In the second stage of the training phase, the start port could be any one of the seven ports, and the target port was one of the six remaining ports.

#### Electrophysiology

**Figure 2.**
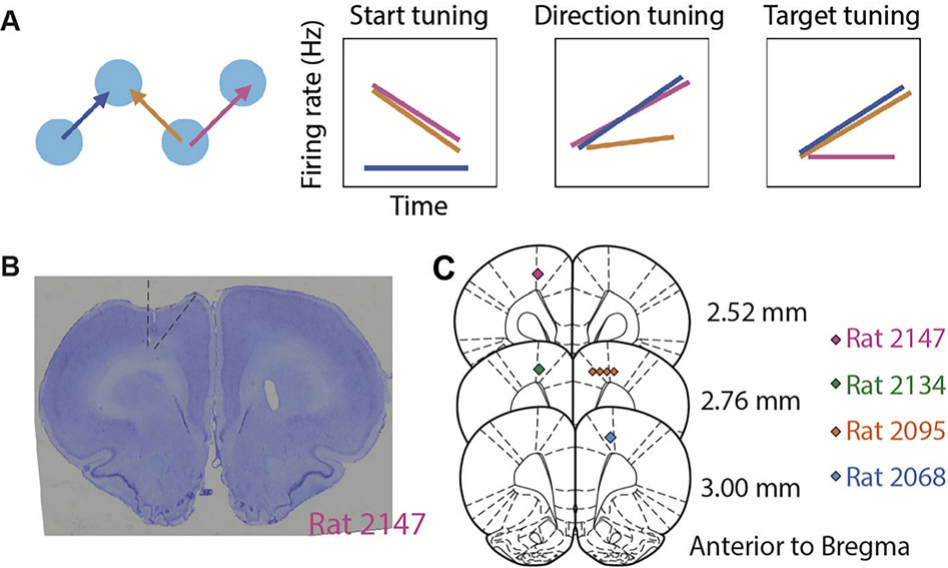
Schematics of the task and histology. ***A***, Three trajectories that can distinguish between tuning to the start position, movement direction, or the target position (left) and the predicted neural activities for each scenario. Among the three trials, the orange and pink trials share the start position, the blue and pink trials share the movement direction, and the blue and orange trials share the target position. If a neuron is tuned to the start position, then the firing rate in the pink and orange trials will be the same, but not for the blue trial. The same logic follows for direction tuning and target tuning cells. ***B***, A coronal section from an example rat (2147) showing the placements of the silicon probes. Dashed lines indicate the estimated area of M2 in this brain section. ***C***, Estimated positions of the tips of the silicon probes at the end of recording presented on coronal sections of the rat brain atlas (Paxinos and Watson, 2004). Lesions were made at the end of all the recording sessions with 200 uA current for 3 s relative to the ground. Colored marks indicate the lesion marks in ***B***, and colors indicate subject ID.

Rats were unilaterally implanted with Cambridge Neurotech microdrives and 64 channel silicon probes. To target the FOF, silicon probes were placed at anteroposterior (AP) and mediolateral (ML) coordinates as following: rat 2068, AP +2.2, ML +1.5; rat 2095, AP +2.0, ML +1.5; rat 2134, AP +2.5, ML −1.5; rat 2147, AP +2.2, ML −1.4 (Fig. 2*B,C*). The probes were placed initially 0.5 mm below the brain surface and were advanced 50–100 µm every 1–4 days, after each recording session. The same neurons could be sampled across sessions.

#### Recurrent neural networks (RNNs)

We trained RNNs with 100 RNN nodes using pytorch. The input to the networks (at each time) was a 100-by-100 pixel image down-sampled to 40-by-40 pixels. The RNN nodes were connected to a two node linear output layer representing coordinates (*x*, *y*). The nonlinearity used in the model was tanh, and stochastic gradient descent was employed during training.

There were four task configurations that differed in the reference frame of inputs and outputs, namely, self-centered input, self-centered output (ego-ego); self-centered input, world-centered output (ego-allo); world-centered input, self-centered output (allo-ego); world-centered input, world-centered output (allo-allo). The networks were trained to take flattened image inputs over 11 time frames (Fig. 10*A,B*). The start position was visible throughout the 11 time frames, and the target position (in the relevant reference frame) was visible briefly at the fourth frame. For the ego-ego and ego-allo networks, the start coordinates were always at the center of the visual frame and the target coordinates were the same as the movement vector. For the allo-ego and allo-allo networks, the start coordinates and target coordinates were both relative to the world frame, and the world frame was always at the center of the visual frame. The input image had a dark background, and the start port, target port, and distractors were denoted as intensities. Specifically, the start port had an intensity of 0.7, the target port had an intensity of 1, and distractors had an intensity of 0.2.

The networks reported the movement vector or the target position at time frame 11 for ego-output and allo-output models, respectively. The target position was in the world frame, whose center was (0,0) and size was 50-by-50 pixels, and the movement vector was the difference between the target position and the start position in the world frame. Making the up-left corner (0,0) did not change the main results.

In the training set, the start port and target port coordinates were randomly generated inside the world frame. In the test set, the start port and target port corresponded to the 24 movement trajectories in the “reference” sessions for Figure 11*A–C* and were randomly generated for Figure 11*D*.

### Statistical analysis

#### Spike sorting

Spike sorting was performed with automatic clustering and manual curation in JRClust. Single units were defined as those with signal-to-noise ratio larger than 5, fraction of inter-spike interval smaller than 1 ms less than 1%, and within-trial firing rate larger than 1 Hz. However, our main results were robust to different single-neuron criteria (Fig. 5).

#### Data inclusion

For a unit to be included in the main figures, the session must have at least eight trials for each of the seven start positions, four or six movement directions and seven target positions. The sessions could be “reference,” “distance,” or sessions from rat 2147. This resulted in 104 sessions and 1,224 single cells in four animals.

In pseudopopulation decoding, we included cells from sessions that had at least eight trials for each of the six movement directions, seven start positions, and seven target positions. The sessions could be “reference” or “distance” sessions. This resulted in 1197 cells from 99 sessions, 3 animals.

Video tracking was available in 58 sessions in 3 rats.

For neuron inclusion criteria for all the main figures, Table 1.

**Table 1.** Criteria for the inclusion of neurons in each analysis

Figure	Panel(s)	*N*	Criteria
Figure 4	*A*	1,224	Single neurons in 4 animals that had at least 8 trials for each condition. Seven hundred and seventy-one neurons from reference sessions, 426 from distance sessions, 27 from rat 2147.
Figure 6	*A–E*	1,197	Single neurons that had at all 6 directions, and at least 8 trials in each group of trial conditions. Seven hundred and seventy-one neurons from reference sessions, 426 from distance sessions. Three rats.
Figure 7	*D* *(black bars)–H*	174	Single neurons that have significant selectivity to start port during the “precue” time window and target port during the “arrival” time window (GLM, both *p* < 0.05), among the 1,224 neurons in Figure 4*A*
Figure 7	*D (white bars)*	289	Single neurons that have significant selectivity to either the start port during the “precue” time window or the target port during the “arrival” time window (GLM, only one *p* < 0.01), among the 1,224 neurons in Figure 4*A*
Figure 7	*I*	202	Single neurons that have significant selectivity to start port during the “pre-cue” time window (GLM, *p* < 0.01), among the 1,224 neurons in Figure 4*A*
Figure 7	*J*	209	Single neurons that have significant selectivity to target port during the “arrival” time window (GLM, *p* < 0.01), among the 1,224 neurons in Figure 4*A*
Figure 8	*A–C*	274	Single neurons that have significant selectivity to direction during the“post-cue” or “go” time window (GLM, *p* < 0.01), among the 1,224 neurons in Figure 4*A*
Figure 9	*E*	199	Single neurons that have the average of *CV R*^2^s larger than 0.05. Effectively from four rats.

Single neurons: SNR > 5, in-trial firing rate >1 Hz, fraction of inter-spike interval less than 2 ms <1%. All the cells are from sessions where the target cue illuminated during the fixation period.

Unless otherwise specified, only correctly performed trials were included in the analysis.

#### Time windows of neural data analysis

In single-neuron level analysis for single spatial variables, we focused on 4 key time windows: *pre-cue*, −300 to 0 ms aligned to target cue onset; *post-cue*, 0 to 300 ms aligned to target cue onset; *go*, 0 to 300 ms aligned to the go sound; *arrival*, −150 to 150 ms aligned to arrival at the target (Fig. 1*B*).

When comparing the pure and mixed selective models (Fig. 9), we used the 0–500 ms time window aligned to the visual cue onset.

Continuous time windows were in sliding windows of 300 ms width and 50 ms step, aligned causally to task events, unless otherwise specified. Causal alignment means that the value at time 0 refers to the neural activity in a time bin between −300 and 0 ms. The neural responses could be aligned to the time of visual target cue onset, the go sound onset, the time of “fixation out” (the time when the nose left the start port, detected by the IR sensors) or target poke (the time when the nose arrived at the target port, detected by the IR sensors).

For warped time windows, there were 3 windows of 300 ms size that spanned −900 ms and 0 ms to the visual cue onset; 3 windows of variable size between the visual cue onset and the go sound; 5 windows of variable size between the go sound and the target arrival; and 3 windows of 300 ms size that spanned the 0 and 900 ms after the target arrival.

#### PETHs

Peri-event Time Histograms (PETH) for example neurons were generated by convolving the spikes on each trial with a causal half-Gaussian kernel of 400 ms standard deviation. Since the kernel was truncated, the effective smoothing was similar to a 200 ms standard deviation full-Gaussian kernel. The kernel was causal so that the smoothed estimate of firing rate at time *t* was contributed by spikes at or before time *t*. Single-trial firing rate estimates were then grouped and averaged over trial conditions as described.

#### Cross-validated R^*2*^*s*

**Figure 3.**
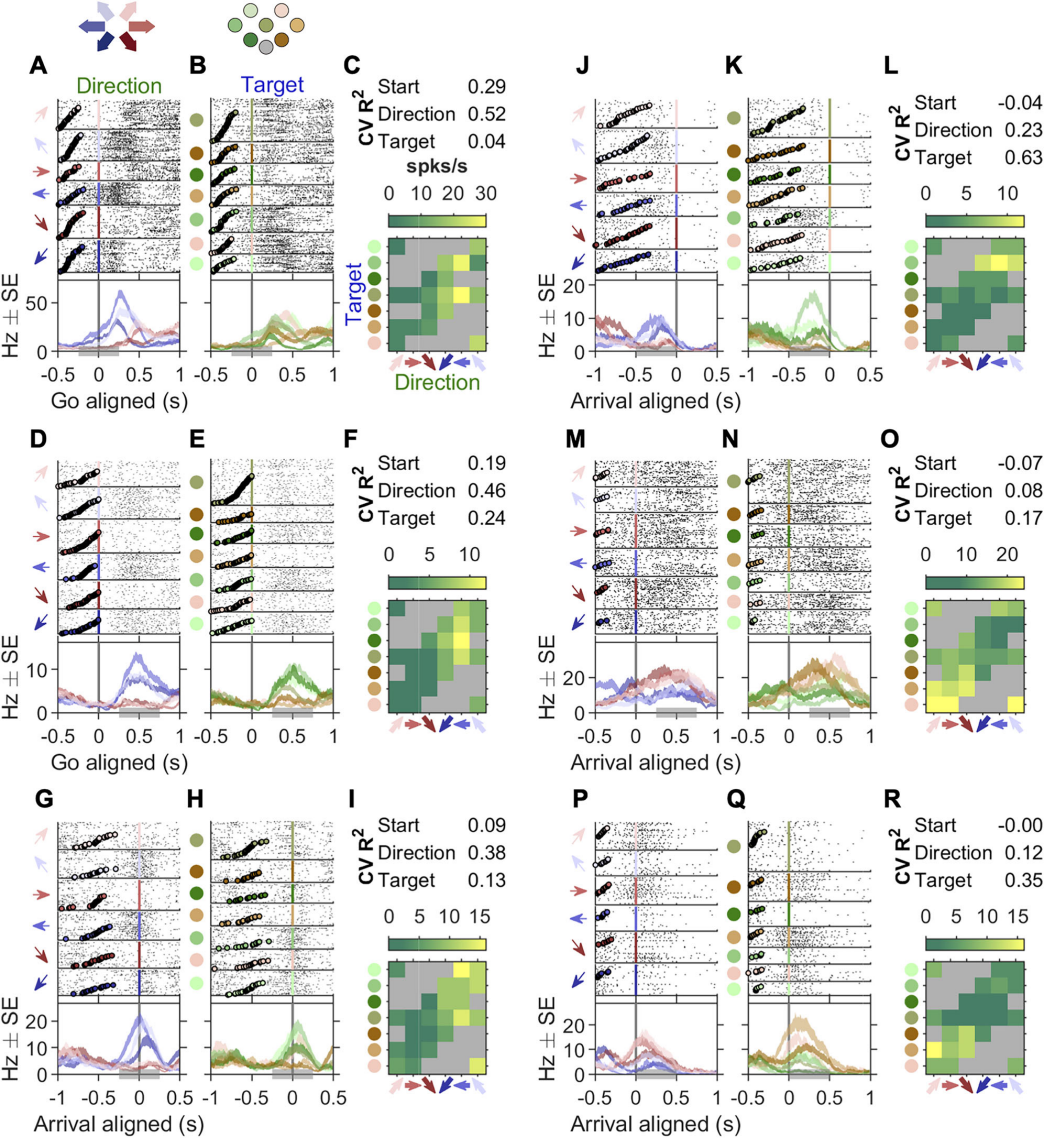
Example neurons with egocentric and allocentric spatial representation. ***A–C***, An example neuron more modulated by the egocentric movement directions than by the allocentric target positions. ***A***, Raster plots and PETHs aligned to the go sound and sorted by movement directions. The top six panels show spike rasters grouped by the six movement directions. Circles in each raster panel indicate the time of the visual cue onset on each trial. The bottom panel shows the PETHs of the spikes. The shaded areas of the PETHs indicate the mean ± s.e. The gray bar at the bottom of the panel indicates the 500 ms time window used to estimate the cross-validated *R*^2^s and the firing rate for each movement trajectory in *C*. ***B***, Raster plots and PETHs of the same cell and same alignment as in ***A***, but sorted by target position. Circles in each raster panel indicate the time of visual cue onset on each trial. ***C***, **CV *R*^2^**. The cross-validated *R*^2^s of three GLMs whose independent variables were the start position, movement direction or target position (See Methods for definition). **Heat map**. The maximum a posteriori estimated firing rate for each movement trajectory, where the prior was a Poisson distribution whose mean was estimated from all trials. Gray squares indicate trajectories (direction-target pairs) that were not included in that session. ***D–F*** and ***G–I***, two more example cells as in *A–C* where direction explained the most variance than start or target positions. Note that in G,H the activity is aligned to arrival. ***J–L***, ***M–N***, and ***P–R***, example neurons whose arrival-aligned activity was more modulated by the target position than direction. Circles in each raster panel indicated the time of the go sound.

The cross-validated *R*^2^s (denoted as *CV R*^2^s in figures) was defined as follows:

$$R^{2}=1-\frac{SS_{\mathrm{res}}}{SS_{\mathrm{tot}}}=1-\frac{\sum_{i=1}^{N}(y_{i}-{\hat{y}}_{i}{)}^{2}}{\text{Var}(y)},$$

where ${\hat{y}}_{i}$ was the predicted mean spike count in the *i*th trial from the test set, and *y*_*i*_ was the observed spike count in this trial. In the best case, *y* is equal to $\hat{y}$, and the cross-validated *R*^2^ is 1. In the worst case, $\hat{y}$ is uncorrelated with *y*, and $\sum_{i}(y_{i}-{\hat{y}}_{i}{)}^{2}/\text{Var}(y)$ can be larger than 1 by chance, thus *CV R*^2^s can be negative. Cross-validated *R*^2^s of the generalized linear models (GLMs) in Figure 3, models in Figure 9 and decoding models in Figure 11*A* were derived from 10-fold cross-validation.

#### GLMs for single-neuron selectivity

We fit the neural spike counts in specific time windows to 3 GLMs with Poisson distributions:

$$\begin{array}{rl} & \text{Spikes}∼\beta_{0}+\beta_{1}X_{\mathrm{start}},\\ & \text{Spikes}∼\beta_{0}+\beta_{1}X_{\mathrm{direction}},\\ & \text{Spikes}∼\beta_{0}+\beta_{1}X_{\mathrm{target}}. \end{array}$$

The spatial variables were all included as dummy variables. Start position had seven levels, direction had six levels, and target position had seven levels.

In the main text, we labeled neurons as “having significant selectivity to a spatial variable.” This significance is derived from the permutation test of the GLM, where the test statistic was the cross-validated log-likelihood of the GLM, against the null hypothesis that the log-likelihood was not significantly different from when trial labels were shuffled. For each spatial variable, the trial labels were shuffled 5,000 times to obtain a distribution of the shuffled model log-likelihoods, then the *p* value of the GLM was the fraction of shuffled log-likelihoods that were greater than the log-likelihood from actual data.

**Figure 4.**
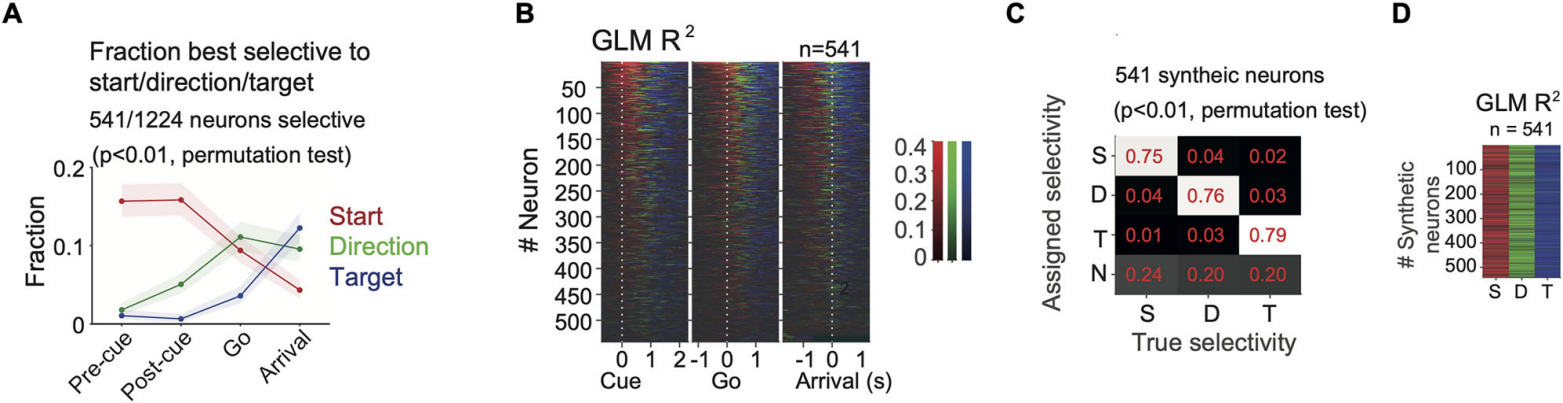
Temporal profile of the fraction of FOF neurons best selective to the start position, direction, and target position. ***A***, The fraction of neurons best selective to the start position (red), the direction, (green) or the target position (blue) in four time windows. Shaded areas indicate the 95% binomial confidence intervals. “Pre-cue,” −300 to 0 ms to visual cue onset. “Post-cue,” 0–300 ms to visual cue onset. “Go,” 0 to 300 ms to go sound. “Arrival,” −150 to 150 ms to target poke as in Figure 1*B*. ***B***, The *R*^2^ of GLMs among same neurons as in A in 300 ms causal sliding windows with 50 ms steps, aligned to the cue onset, go sound or target arrival. At each time point, the color represents the variable with the largest *R*^2^ and the saturation represents the *R*^2^ value. Neurons were sorted by the total mass of the *R*^2^s of start position, direction and target position for the three alignments respectively. ***C***, Fraction of synthetic neurons most selective to each generative spatial variable. Each column represents 541 synthetic neurons that were designed to be selective to start position (S), direction (D), or target position (T), or nonselective (N). The vast majority of errors were false negatives (where a neuron was incorrectly labeled nonselective). ***D***, The *R*^2^s of GLMs in synthetic neurons with specific spatial selectivity. Each column was a group of 541 synthetic neurons selective to start, direction or target, respectively. The color indicated the *R*^2^ of the model with the maximum *R*^2^, with the same scale as in *B*.

The best selectivity of a neural response (Fig. 4*A*) was assigned to the spatial variable with the smallest *p* value of the GLM. When there were ties in *p* values, we additionally compared the cross-validated log-likelihoods, and the best selectivity was assigned to the spatial variable with the largest sum of log-likelihoods. The reason for possible ties is that we are using permutation tests with 5,000 permutations.

**Figure 5.**
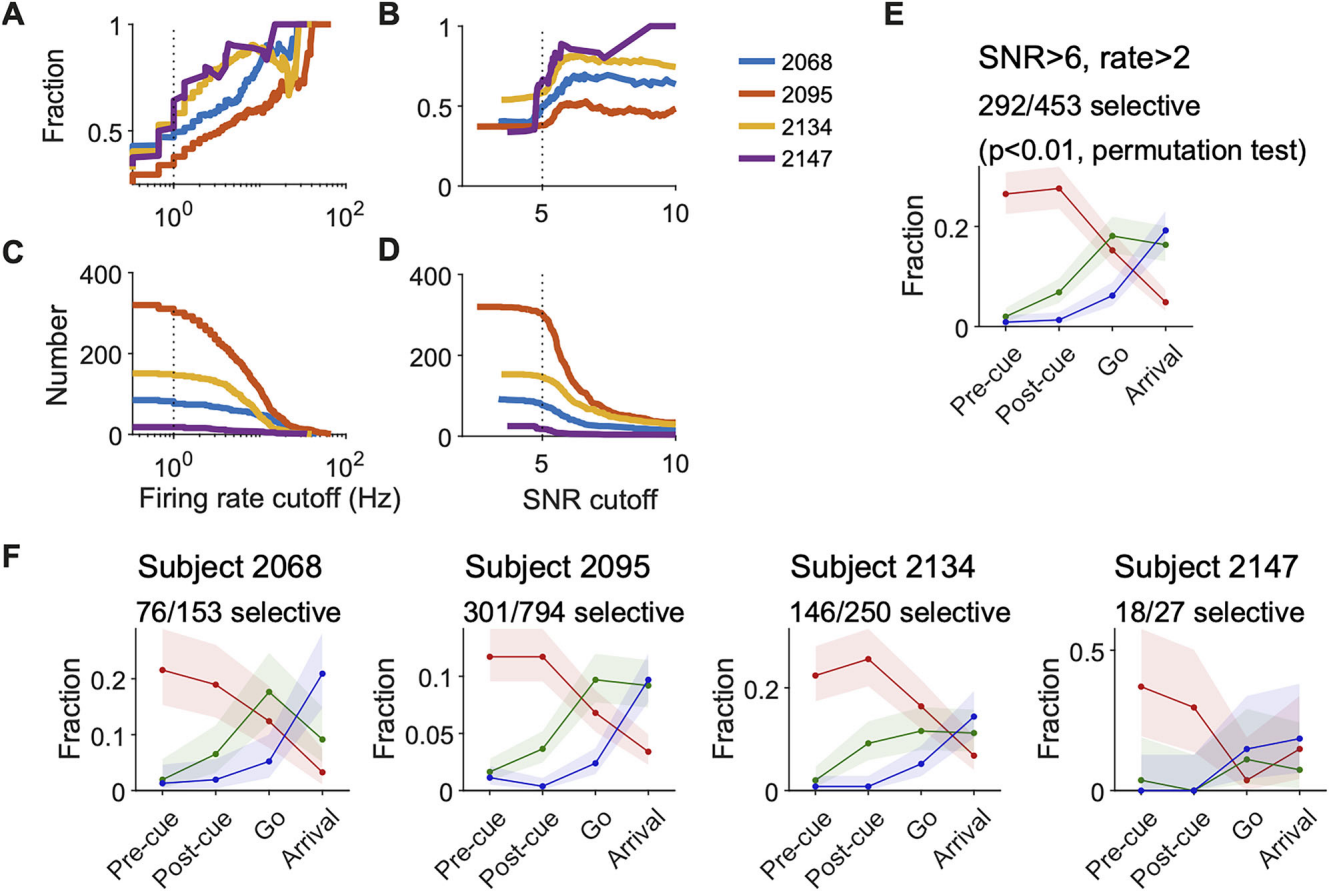
The sequential encoding of start position, direction, and target position was consistent across single-neuron selection criteria and across subjects. ***A***, The fraction of units with significant spatial selectivity at different cut-off criteria for the in-trial firing rate. The cutoff of the signal-to-noise ratio (SNR) of the waveform was fixed at 5. A unit is characterized as having significant spatial selectivity if any one of the three GLMs had *p* < 0.01 (permutation test) for any one of the four time windows (“pre-cue,” “post-cue,” “go,” or “arrival”). ***B***, The fraction of units with significant spatial selectivity at different cut-off criteria for the SNR of the waveform. The cut-off criteria for in-trial firing rate was fixed at 1 Hz. ***C***, The number of units with significant spatial selectivity at different in-trial firing rate cutoff. ***D***, The number of units with significant spatial selectivity at different SNR cutoffs. ***E***, Similar to Figure 4*A*, but the cut-off criteria is SNR >6 and in-trial firing rate >2 Hz instead of SNR >5 and firing rate >1 Hz. ***F***, Similar to Figure 4*A*, but for each subject.

The cross-validated log-likelihoods were calculated with leave-one-out cross-validation. We assumed spike counts to follow the Poisson distribution, and the cross-validated log-likelihood is defined as follows:

$$LL=\sum_{i=1}^{N}\log\;(P(y_{i}|\hat{y_{i}})),$$

where $P(y_{i}|\hat{y_{i}})$ denote the probability of observing the spike count on the *i*th trial being *y*_*i*_, given the predicted mean spike count being $\hat{y_{i}}$. *N* denote the number of trials.

In Figure 4*B*, the *R*^2^s were derived from GLMs without cross-validation. Only the model with the largest *R*^2^ was plotted with the corresponding color at each time point. Both the best GLM measurement and the *R*^2^ measurement for the relative strength of spatial selectivity were robust to the task-induced correlation between spatial variables, which was verified with synthetic data (Fig. 4*C,D*).

#### Spatial preference of single neurons

The preferred direction of a neuron in a specific time window was defined as the direction of the weighted vector sum of the coordinates of the 6 movement directions, where the weight for each direction was the mean firing rate in that direction. Horizontal directions were defined as directions between $-45^{\circ }$ and $45^{\circ }$ around the horizontal directions, and so was for vertical directions. Downward directions were directions that were $-90^{\circ }$ to $90^{\circ }$ around the downward direction, and so was for upward directions. The preferred position of a neuron at a specific time window was simply denoted as the port with the highest mean firing rate.

#### Start-target tuning correlation

The start-target tuning correlation in Figure 7*D* was defined as the Pearson correlation between the tuning curve for start position in the “pre-cue” window and target position in the “arrival” window:

$$\rho =\text{cov}(FR_{\mathrm{start}},FR_{\mathrm{target}})/\sigma (FR_{\mathrm{start}})\sigma (FR_{\mathrm{target}}),$$

where $FR_{\mathrm{start}}$ is a vector where each element is the mean firing rate for a specific start position, and $FR_{\mathrm{target}}$ is a vector where each element is the mean firing rate for a specific target position. Similarly, we calculated the start-target tuning correlations for different pairs of time windows and the tuning correlation between time *t*_*i*_ and *t*_*j*_ for the same variable. The time windows were warped to align to the time of the visual cue, the go sound and the target poke. The 300 ms “pre-cue” and “arrival” time windows were preserved. For each time, we randomly split the trials in the session into two sets and then we used start tuning in the first set versus target tuning in the second set, and vice versa, and then took the average of the two correlation coefficients.

The tuning correlation was subject to the Fisher-z transformation, and then tested against the null hypothesis that the mean value across a specific neural population was not significantly larger than zero. The *p* value indicated the fraction of mean lying below zero among 10^4^ bootstraps, and was corrected with Bonferroni correction.

#### Timing of start-target switching

In Figure 7*I*, spike counts in causal 300 ms time windows of 50 ms steps were fit to the Poisson GLM of either start or target position. The *R*^2^s of each GLM across time was smoothed with a moving average kernel with the size of three bins, and then the start GLM *R*^2^ was subtracted by the target GLM *R*^2^. The time of switching from start encoding to target encoding was defined as the first time window where $R_{\mathrm{start}}^{2}-R_{\mathrm{target}}^{2}<0$, after the positive peak of $R_{\mathrm{start}}^{2}-R_{\mathrm{target}}^{2}$.

#### Mixed selectivity

To detect mixed selectivity for each neuron, we compared between four models of the neural firing rate.

In the Gaussian direction tuning model, the firing rate was a Gaussian function centered by the preferred direction, defined as in the “Spatial preference of single neurons” section in Methods.

$$f(\theta )=b_{0}+b_{1}\frac{1}{\sigma \sqrt{2\pi }}\exp\;\left(-\frac{\theta^{2}}{2\sigma^{2}}\right).$$

In the start position plane model, the firing rate was modulated linearly by the horizontal and vertical coordinates of the start position.

$$f(x,y)=b_{0}+b_{1}x+b_{2}y.$$

In the gain-field model, the firing rate was a multiplicative combination of a Gaussian tuning centered by the preferred direction and a start position modulation.

$$f(\theta ,x,y)=b_{0}+(b_{1}x+b_{2}y)\frac{1}{\sigma \sqrt{2\pi }}\exp\;\left(-\frac{\theta^{2}}{2\sigma^{2}}\right).$$

In the additive model, the firing rate was an additive combination of a Gaussian tuning centered by the preferred direction and a start position modulation.

$$f(\theta ,x,y)=b_{0}+(b_{1}x+b_{2}y)+\frac{b_{3}}{\sigma \sqrt{2\pi }}\exp\;\left(-\frac{\theta^{2}}{2\sigma^{2}}\right).$$

In these functions, *f* was the firing rate, *θ* was the movement direction relative to the preferred direction, and *x* and *y* are the horizontal and vertical coordinates of the start positions in the port wall. *b*_0,1,2,3_ and *σ* were fit for each model with maximum likelihood estimation assuming Poisson spike counts, using the Matlab *fmincon* function. The models were fit with 20-fold cross-validation.

For each pair of models, we compared the cross-validated *R*^2^s among neurons whose average of cross-validated *R*^2^s of the four compared models was larger than 0.05. Significance level was tested against the null hypothesis that the mean difference between the Fisher-z transformed cross-validated *R*^2^s in the *x*-axis model and the *y*-axis model was not significantly larger than zero, quantified as the fraction of the mean value smaller than zero among 10^5^ bootstraps.

For a given movement direction, the target position coordinates were simply an additive translation of the start position, thus the start position and target position modulations were the same in the gain-field model and the additive model.

#### Pseudopopulation decoding

Pseudopopulations were constructed separately when decoding each spatial variable. For example, when decoding the start position, we constructed the pseudopopulation by resampling trials for each start position. For each neuron, trials were randomly split into 2 folds for each condition of the spatial variable, and 32 trials were resampled for each fold in each condition, yielding 224 trials per fold for position decoding and 192 trials per fold for direction decoding. These resampled trials were termed “pseudo-trials.” Pseudo-trials were resampled randomly with a different seed for each neuron, so as to remove trial-by-trial correlations between neurons in the same session.

We included single neurons from sessions that had at least 8 trials for each of the 7 start positions, 6 directions and 7 target positions (1197 neurons, 99 sessions, 3 rats). In each pseudopopulation, neurons were also resampled with a different seed, so that only about 63.2% of the neurons were included in each pseudopopulation. We generated 100 pseudopopulations for the decoding each spatial variable.

The error of decoding was defined as the Euclidean distance between true and predicted coordinates. The goodness of prediction was measured as the mean error between the predicted and the actual coordinates across all the pseudo-trials in that pseudo-session. For start and target position, we removed the trials with [0 0] coordinates from the quantification of decoding accuracy. This is because predictions from unsuccessful decoding tend to cluster at the [0 0] coordinates, so decoding accuracy for the [0 0] coordinates in positions (i.e., the central port) will always seem “good.”

**Figure 6.**
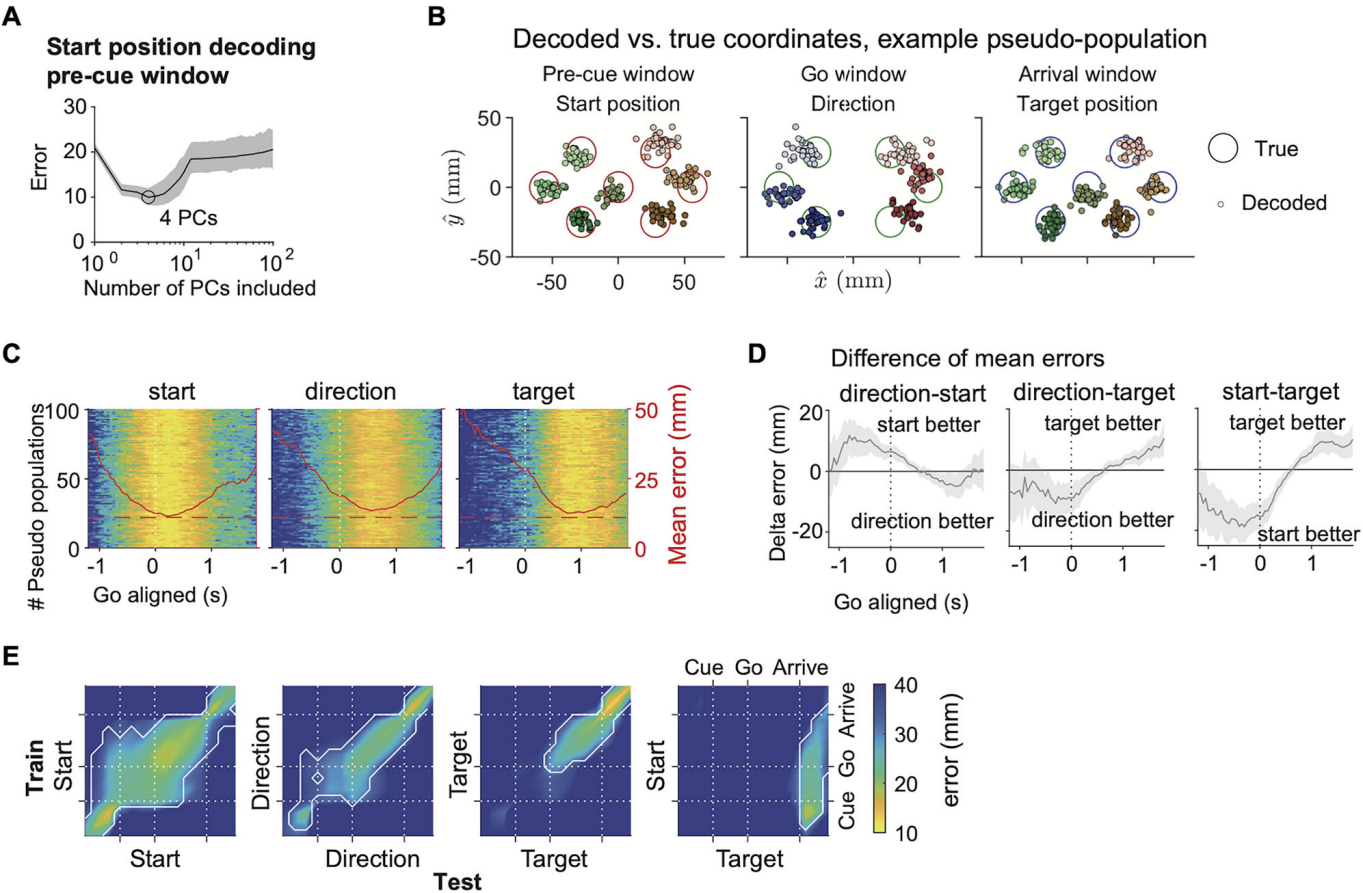
Temporal profile of start position, direction, and target position decoding from the FOF pseudopopulation. ***A***, Number of principle components included versus the accuracy of pseudopopulation decoding for the start position, using spike count data in the pre-cue time window. Thin line and shaded areas indicated the mean and the 5% and 95% intervals in 100 pseudopopulations with neurons resampled with replacement. Error is defined as the Euclidean distance between the predicted and the actual coordinates. ***B***, Decoded coordinates of start position, movement vector, and target position in an example pseudopopulation. Each small circle indicates the predicted coordinates in a pseudo-trial, and the color indicates the pseudo trial class. Each large circle indicates the coordinates and the radius of a port (11 mm). ***C***, Decoding errors for each pseudopopulation across time aligned to the go sound. Each row is a different pseudopopulation, and the color indicates decoding error following the color bar in *E*. Red solid lines indicate the mean errors across the 100 pseudopopulations. Red dashed lines indicate the radius of the ports. ***D***, Mean ± s.d. of the difference of decoding errors between two spatial variables across the 100 pseudopopulations. Positive difference indicates the better decoding of the second variable, and vice versa. ***E***, Decoding errors with cross-window decoding. Colors of the heat maps indicate the mean Euclidean distance between the decoded and true spatial coordinates, averaged across 100 pseudopopulations. The decoders were trained at one time window and tested at another. In the last panel, the multivariate linear model was trained for start position and decoded for target position. Contours, *p* < 0.01 (extreme pixel-based test). Pseudopopulations were constructed from neurons with at least eight trials for each of the six directions, seven start positions and seven target positions (*n* = 1, 197).

Decoding was performed with multivariate regression models (*mvregress* function in Matlab Statistics and Machine Learning Toolbox). The spike counts of the training set and the test set were first combined, *z*-scored, and applied to principle component analysis, and then split again for training and decoding. We found that the cross-validated decoding accuracy was the best when including only the first four principal components (PC) when decoding start position from neural activity in the “pre-cue” time window (Fig. 6*A*), so we used four PCs for all subsequent pseudopopulation decoding.

**Figure 7.**
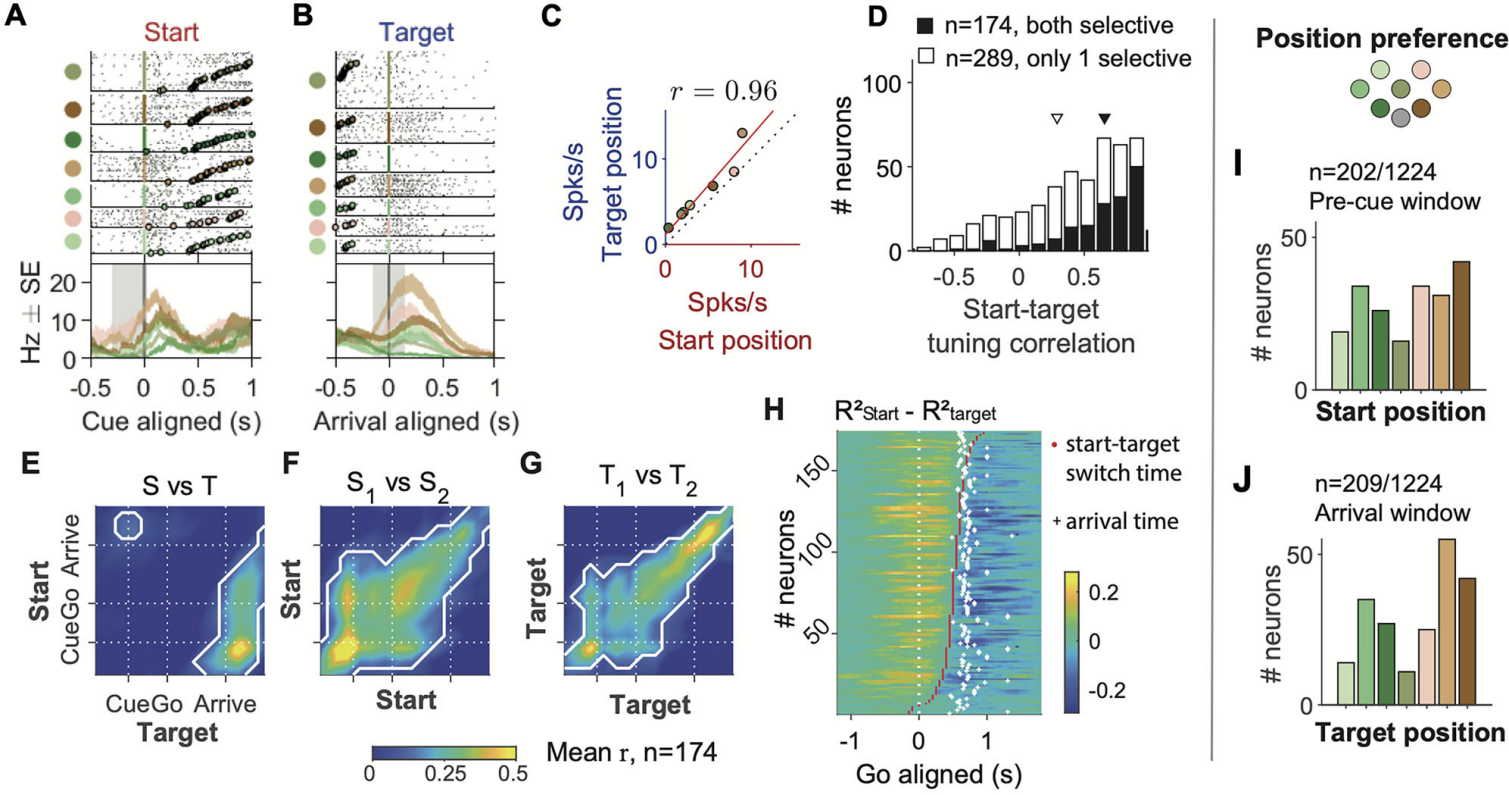
FOF neurons were tuned to specific positions. ***A***, Raster plots and PETHs of an example neuron aligned to the cue, grouped by start position. The shaded gray area indicates the time window to calculate the *x*-axis firing rate in C. The neuron was the same one as in Figure 3*P–R*. ***B***, the same neuron aligned to target arrival, grouped by target position. ***C***, The correlation between start position tuning and target position tuning in the example neuron. Red line denotes the total least-square fit. *r* denotes the Pearson correlation between start and target tuning, which was termed start-target tuning correlation. ***D***, The distribution of the start-target tuning correlation among start and (or) target selective neurons. Black bars are for neurons selective to both start position and target position, and white bars are for neurons selective to only one of the two variables (mean, [95% CI]: 0.66, [0.61, 0.70] for both selective and 0.29, [0.25, 0.34] for only 1 selective). Triangles indicate the means of the two groups. ***E***, The start-target tuning correlation in warped time windows aligned to the visual cue, the go sound and the arrival, averaged across neurons with both start and target selectivity (*n* = 174). The white contours indicate the areas where correlation is significantly larger than 0 (*p* < 0.05 after Bonferroni correction). Different from *C* and *D*, these correlations were calculated between start tuning in half of the trials and target tuning in the other half of trials and vice versa, and then averaged. ***F***, Similar to *E*, but for the mean Pearson correlation between pairs of time windows for start position tuning in one half of trials versus the start tuning in the other half. ***G***, Similar to *F*, but for target position tuning. ***H***, Time of transition from start position coding to target position coding in the 174 neurons. The color of the heat map indicates the difference between *R*^2^s of the start position GLM and the target position GLM, calculated at causal sliding 300 ms time windows of 50 ms step size aligned to the go sound. The red dots indicate the time of switching from $R_{\mathrm{start}}^{2}$ higher to $R_{target}^{2}$ higher. The white crosses indicate the averaged time of target poke for that session. ***I***, The number of neurons preferring each start position in the “pre-cue” time window, among cells that had significant start position selectivity in the “pre-cue” window (*p* < 0.01, permutation test for the start position GLM). ***J***, The number of cells preferring each target position in the “arrival” time window, among cells that have significant target position selectivity in that window (*p* < 0.01, permutation test for the target position GLM).

In Figure 6*B*, multivariate regression models were trained and tested with spike counts in 3 key time window as indicated in the plot (see “Key time windows” section for details). The plots showed the result from one example pseudo-session, the same pseudo-session as the first row in Figure 6*C*. In Figure 6*C*, the time windows were 300 ms width with 50 ms step, causally aligned to the go sound. Each row was a different pseudo-session. Each row of the heat map was a pseudopopulation with a different resampling of neurons, and the color indicated the mean error with two-fold cross-validation. In Figure 6*D*, the mean error of one spatial variable was subtracted by the mean error of another spatial variable in each pseudopopulation, termed the Delta error, which represented the relative goodness of decoding between the two spatial variables. The distribution of Delta errors of the 100 pseudopopulations was then illustrated with the mean and standard deviation.

In Figure 6*E*, models were trained at one time window and tested at another also with two-fold cross-validation. In the last panel, a multivariate regression model was trained to decode start position coordinates from the start position pseudopopulation data, and then tested its decoding of target position coordinates from the target position pseudopopulation data. The heat map showed the average of mean errors across 100 pseudopopulations. *P* values for multiple comparisons were obtained with extreme pixel-based permutation test (Cohen, 2014). One dummy heat map was generated for each pseudopopulation by shuffling trial labels, and the maximum of each heat map was gathered to construct a null distribution. The white contours enclosed the area in which the averaged mean error was smaller than the minimum value of this distribution (*p* < 0.01).

#### Head position estimation and correlation with movement direction

We estimated the coordinates of the head, the ears and the hip of the rat in the video frames using DeepLabCut (Mathis et al., 2018). Coordinates were in the unit of pixels. The head position was approximated as the Intan chip plugged on the animal’s head. For continuous sampling of body coordinates aligned to task events, we linearly interpolated to achieve the sampling rate of 100 frames per second. In Figure 1*J*, we plotted the horizontal head coordinates in an example session during center-out trials aligned to the visual cue onset.

For each session, we fitted the horizontal head position coordinates in the video pixel space to a linear mixed effect model: head_*x*_ 1 + direction + (1|start poke). The directions and the start pokes were included as factors. We then plotted the distribution of the *p*-value of this model for each session across time in Figure 1*K*.

#### Representation similarity analysis (RSA) analysis on RNN data

To examine the representation similarity between the hidden units in the RNN and the real neurons, we focused on the hidden unit activity at frame 3, 7 and 11. These frames corresponded to real neural activity during the pre-cue, post-cue and go time windows in the task, respectively. We first computed the first 4 principle components of the hidden unit activity and real neuron activity separately, under the 24 movement trajectories. We then constructed the representation dissimilarity matrix (RDM) for the two datasets by calculating the correlation coefficients for each pair of trial configurations. Finally, we computed the Pearson correlation coefficient for the linearized lower-triangular matrix for the two RDMs, as the measurement for the similarity between neural representation of the RNN and the real neurons.

#### Synthetic data

To verify the reliability of our model comparison approaches, we performed the same analysis on synthetic data which were known to have specific spatial tuning as on real neurons. Synthetic neurons were generated to have a known spatial tuning, to match the tuning curve and the overall firing rate of a real neuron, and as if the behavior was sampled from a real session.

To synthesize a neuron that had a specific tuning function, we first fit the spike counts of a real neuron to that tuning function, so as to obtain the predicted firing rate at each trial condition. We then randomly selected a real session and predicted the firing rate from the tuning function and the behavioral data in that session. We finally used a Poisson random process to generate spike counts that followed these predicted firing rates.

In the synthetic data for GLMs, we only used neurons that have significant selectivity to that GLM (*p* < 0.01) to generate synthetic data. In synthetic data to compare between pure and mixed selectivity models, we used all neurons to generate synthetic data.

Following stages of analyses was identical for synthetic data and real data. If our methods were reliable, then the synthetic data designed to have a specific functional form would be best fit by the same functional form.

#### Statistical analysis softwares

Statistical analysis for the behavioral and neural data, as well as the RSA analysis was performed in Matlab unless otherwise specified. Statistical analysis for the decoding of spatial variables from hidden units of the RNNs were performed in Python.

## Results

We trained rats to perform visually guided orienting movements to multiple directions and multiple target positions (Fig. 1*A,B*). The training apparatus consisted of a vertical port wall with seven operant ports and an additional port for reward delivery. Each trial began with illuminating the yellow and blue LEDs around a “start port,” which was randomly chosen from one of the seven operant ports. Rats fixated in the start port until a go sound. The start port LEDs extinguished at the beginning of fixation, and the target port was illuminated with blue LEDs shortly after fixation onset with 0–0.29 s delay. After the go sound, rats withdrew from the start port and moved to the target port. The target port LEDs extinguished once the animal arrived at the target port or when the animal poked in another port in error. If the rat poked in the correct port, the water delivery port LED illuminated, a “correct” sound was played, and reward could be collected at the reward port. If the rat poked into the wrong port, an “error” sound was played and there was no reward. A trial was considered incomplete if the animal did not poke into any port after the start port within 15 s. Unless otherwise specified, incomplete trials and fixation violations were not included in analyses.

The task involved six movement directions and seven start and target positions. Throughout the paper we indicate self-centered movement directions with a blue-red (left/right) and dark-light (down-up) color scheme (Fig. 1*C*). For world-centered positions we use a green-orange (left/right) and dark-light (down-up) color scheme (Fig. 1*D*). Out of the possible 42 movement (7 start ports × 6 target ports), we selected 30 movement trajectories to include in the task (Fig. 1*E*). Each session only involved a subset of 16–24 trajectories out of the 30 (see Methods for details), and data from these sessions are pooled unless indicated.

Rats performed 318.89 ± 7.76 (mean ± s.e.) trials in each 1.5 h recording session. As expected from a visually guided task, performance was good (% Correct = 94.03%±0.53%, mean ± s.e., *n* = 104 sessions; Fig. 1*F*). Most of the errors were made into the same left/right direction relative to the instructed direction (Fig. 1*G*; % Same = 93.55%±0.19%, mean ± s.e.), that is, the most errors were made upper or lower relative to the instructed direction. Lower errors were significantly more common than upper errors in two of the four subjects (Fig. 1*H*; subject 2068, *t*(35.95) = 1.46, *p* = 0.154; subject 2095, *t*(76.53) = 12.08, *p* = 2.11 × 10^−19^; subject 2134, *t*(59.90) = 4.82, *p* = 1.01 × 10^−5^; subject 2147, *t*(6.11) = −1.29, *p* = 0.26, Welch’s *t*-test). The fixation period was between 0 and 1.2 s among the four animals (Fig. 1*I*).

The heads of the rats were kept still during the fixation period (Fig. 1*J*). To test whether subtle shifts in the head position during fixation could predict upcoming choice, we regressed the horizontal head position to the upcoming movement direction and plotted the *p*-value across time. For most sessions, *p* was larger than 0.05 until 0.2 s after the go sound, indicating that the head position during fixation was not significantly correlated with the future movement direction (Fig. 1*K*).

### FOF neurons were tuned to self-centered movement directions and world-centered head positions

We reasoned that if preparatory activity in the FOF represents self-centered movement direction, then neurons would fire similarly during trials with the same direction. Similarly, if the preparatory represents the upcoming world-centered target position, then neurons would fire similarly during trials into the same target position (Fig. 2*A*). Movement trajectories in our task allowed us to dissociate these different scenarios.

We recorded 1,224 single neurons in the FOF from 104 sessions in four rats (Fig. 2*B,C*). As expected from previous studies (Erlich et al., 2011), there were neurons strongly tuned to the self-centered movement direction, during the planning phase (Fig. 3*A–C*) or the execution phase (Fig. 3*D–F, G–I*). Interestingly, there were also neurons more strongly tuned to the world-centered target position than self-centered movement directions upon target arrival (Fig. 3*J–L, M–O, P–R*). When firing rates were conditioned on both direction and target position, many neurons seemed to code the conjunction of position and direction (Fig. 3*C,F,I,O,R*). We will address conjunctive coding in later sections. First, we will ask which spatial variable do FOF neurons most strongly encode at each phase of the trial?

### FOF neurons encoded self-centered movement plans prior to world-centered target positions

From visual examination of neural activity, there was considerable heterogeneity in task variable tuning as well as the temporal dynamics of tuning across the trial. To get a holistic view of the temporal dynamics of spatial tuning in single neurons, we fit the spike counts of each neuron in four 300 ms time windows to the three Poisson GLMs, one for each variable of interest (start position, direction or target position). The time windows were: “pre-cue,” −300 ms to 0 ms aligned to the visual cue onset; “post-cue,” 0–300 ms aligned to the visual cue onset; “go,” 0 to 300 ms aligned to the go sound; “arrival,” −150 to 150 ms aligned to the target arrival (Fig. 1*B*). We fit three separate GLM instead of a single GLM including all variables because that design matrix would not be full rank due to the linear relationship between variables: target = start + direction.

Of the 1,224 neurons, 541 (44%) were selective to one or more task variables (start position, direction or target position) in at least one time window. Due to the correlations between variables in the task, a neuron with strong pure start tuning could be found to be spuriously tuned for direction since, for example, for trials starting at the bottom-left port there are three possible movements, two of which are rightward. To address this potential confound, we estimated which of the three task variables *best* explained the neural activity in each time window. Most neurons were best tuned to the start position in the pre-cue time window. After the visual cue onset, direction tuning increased, whereas target position tuning emerged later and peaked at the arrival time window (Fig. 4*A*). Thus, the neurons in FOF encoded the egocentric movement direction before the allocentric target position, even though the appearance of a visual target cue provided information about the movement direction *at the same time as* the target position. We then extended the analysis by sliding time windows aligned to the visual cue, go sound or target arrival, and the same temporal trend was captured by the *R*^2^s of the corresponding GLMs across time (Fig. 4*B*). Note the clear visual transition from red (start) to green (direction) to blue (target) over time.

To validate our method of assigning which single task variable best explained neural activity, we generated synthetic neurons that had a specific spatial tuning with matched firing rate and tuning strength to real neurons, and found that more than 70% of synthetic neurons were assigned to the generative variable. The majority of errors were in the failure of detecting any selectivity, rather than errors in classification (e.g., incorrectly labeling a “start” neuron as “untuned” rather than a “direction” or “target” neuron; Fig. 4*C*). If we ignore false negatives, then the correct assignment was above 90%. The correct assignment rate in the three synthetic data groups were not significantly different (*χ*^2^(2, *N* = 1623) = 1.65, *p* = 0.439). The *R*^2^s of the GLMs were also verified to capture spatial selectivity, in that synthetic start neurons had high *R*^2^ for start and likewise for synthetic direction and target neurons (Fig. 4*D*).

The findings about the relative time course of selectivity were robust to different inclusion criteria for neurons, such as firing rate and signal-to-noise ratio (Fig. 5*A–E*). The results were also reliable across the rats in the study, with 3/4 rats clearly showing an increase in direction tuning earlier than target position tuning (Fig. 5*F*).

In the single-neuron analysis above, we found that at the time of the go signal, more neurons were best described as “direction” rather than “target” neurons. However, a neuron that is best described as a “direction” cell, might also have target information. To investigate the relative emergence of task variable encoding without the lens of “best single variable” categories, we examined the geometry of spatial representation on a continuous scale at the population level using pseudopopulation decoding. To generate pseudopopulations, we pooled all the neurons across sessions where there were at least 8 trials for each start position, direction or target position. This criteria restricted our dataset to 1,197 neurons from 99 sessions. To prevent a few neurons in the population from dominating the decoding, we randomly resampled the neurons with replacement to construct 100 pseudopopulations. We decoded the *x* and *y* coordinates of each task variable using multivariate linear decoders with two-fold cross-validation, from the first 4 principle components of the pseudopopulation activity in 300 ms time windows (Fig. 6*A*). The errors, defined as the Euclidean distance between the predicted and the actual spatial coordinates, were as small as the radius of the port (around 11 mm) at the best time window for each spatial variable (Fig. 6*B,C*). Put in other words, the geometry of both the movement directions and the port positions were embedded in a linear subspace of the FOF activity.

Pseudopopulation decoding accuracy also demonstrated the sequential encoding of start position, movement direction and target position. The relative goodness of decoding between two spatial variables was quantified as the difference between the two mean decoding errors (Fig. 6*D*). Start position was decoded better than movement direction or target position in the early phase of a trial. At the time of the go cue, direction decoding was better than target decoding (*p* = 4 × 10^−4^, permutation test with 5,000 shuffles). Then target decoding became better than direction decoding at 0.65 s after the movement onset. We examined the stability of representations by training decoders at one time and then testing them at other times (Fig. 6*E*). We found start position tuning was stable across much of the trial. Direction coding was stable from cue onset to shortly after the go cue, but then switched modes during the movement. This is consistent with results from mouse motor cortex (Inagaki et al., 2022). Target decoding emerged after the go cue and was not particularly stable across the movement period. Interestingly, decoders trained with start positions during fixation could accurately decode target positions around target poke, suggesting a consistent coding for the current head position throughout the trial.

### Single FOF neurons were tuned to specific port positions regardless of trial epoch

We reasoned that the stable position coding (rightmost panel in Fig. 6*E*) in the pseudopopulation could be supported by single neurons that had tuning for a specific position regardless of the time in the trial. For example, a neuron that fired strongly for the top-left port during the early fixation period, also responded strongly at the end of the trial when the top-left port was the target. Indeed, we found neurons whose tuning for the start port during fixation was similar to the tuning for the target port when the head arrived at the target (Fig. 7*A,B*). We quantified this consistency using the Pearson correlation between start position tuning in the “pre-cue” window and target position tuning in the “arrival” window, and denoted this as the “start-target tuning correlation” (Fig. 7*C*). Among neurons selective to both the start and the target position (*p* < 0.05 for both the start and the target GLMs, *n* = 174), the mean start-target tuning correlation was significantly positive (0.66, [0.61,0.70], mean, [95% CI], *p* = 2 × 10^−4^, permutation test with 10,000 replicates; Fig. 7*D*).

To investigate the temporal dynamics of the correlation across the trial, we computed the start-target tuning correlation for each neuron between one time window and another. Among neurons selective to both the start and the target position (*p* < 0.05 for both the start and the target GLMs, *n* = 174), the mean correlation between start position tuning early in the trial and target position tuning around target poke were positive (Fig. 7*E*). For comparison, in the same group of neurons, the peak of the mean start-target tuning correlation among these neurons was 0.555, on the same scale as the target-target tuning correlation, which was 0.579, suggesting highly consistent start and target position tuning (Fig. 7*F,G*).

The consistency of start position and target position tuning were not limited to strongly tuned neurons. Among all the neurons with spatial selectivity (*p* < 0.05 for any one of the GLMs, *n* = 808), the start tuning correlation, the target tuning correlation, and the start-target tuning correlation were all positively correlated (start tuning correlation and target tuning correlation, *r*(806) = 0.383, *p* = 4.49 × 10^−7^; start tuning correlation and start-target tuning correlation, *r*(806) = 0.354, *p* = 2.85 × 10^−6^; target tuning correlation and start-target tuning correlation, *r*(806) = 0.270, *p* = 5.56 × 10^−4^; Pearson correlation). In other words, neurons tuned to the start position were more likely tuned to the target position, and the start and target tunings were more likely to be consistent. Collectively, these evidence suggested that single FOF neurons tracked the current head position.

One might notice the correlation between start position tuning late in the trial and target position tuning early in the trial (Fig. 7*E*). This was due to the correlation between start and target positions in the task, which led to the weak “mirroring” of the strong early-start to late-target correlation.

Start position encoding transitioned to target position coding mainly during the movement period (Fig. 7*H*). We fit the neural spike counts across time to the start and the target GLMs, and defined the time of transition as the time when the *R*^2^ of the start position GLM first became smaller than the target position GLM (see Methods for details). For most of these neurons, the switch time was between the go sound and the target poke arrival time.

The numbers of neurons preferring each start position and target position spanned across all the ports (Fig. 7*I,J*). The preferred positions were not uniformly distributed. There were more neurons with preferred start positions (*χ*^2^(6, *N* = 202) = 17.35, *p* = 0.009) and target positions (*χ*^2^(6, *N* = 209) = 48.39, *p* = 9.86 × 10^−9^) at the most leftward and rightward ports. Consistent with the current head position coding, the distribution of the preferred start position among start position selective neurons were not significantly different from the distribution of preferred target position among target position selective neurons (*χ*^2^(6, *N* = 411) = 9.67, *p* = 0.139; Fig. 7*I, J*).

### FOF neurons represented movement directions asymmetrically

**Figure 8.**
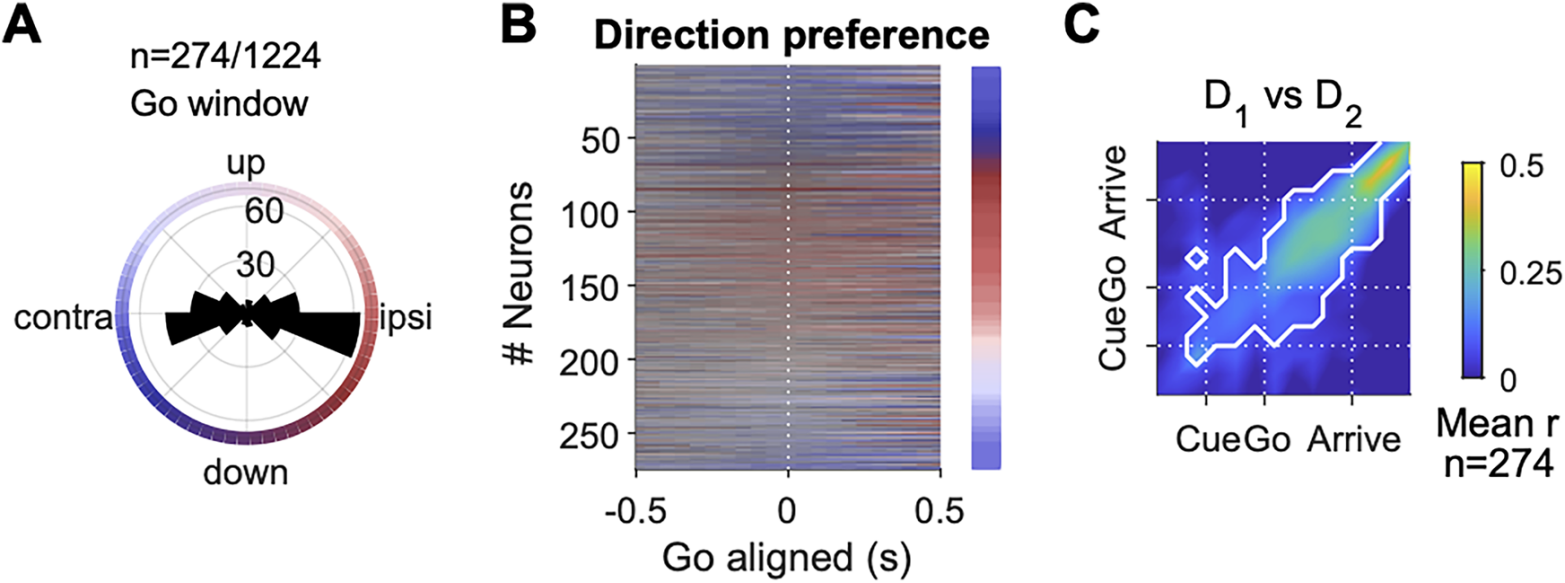
Direction preference of FOF neurons. ***A***, The distribution of preferred direction in the “go” time window (0–300 ms after the go sound) among 274 cells that had significant direction selectivity in either the “post-cue” or “go” time windows (*p* < 0.01, permutation test for the GLM). Note, that the color scheme here represents ipsilateral versus contralateral to the recording side in the horizontal direction (as opposed to right vs left in other figures). ***B***, The preferred direction of neurons in *A* in causal sliding windows aligned to the go sound (50 ms step size, 300 ms bin size). The color indicates the preferred angle (as in *A*) and the saturation indicates the relative amplitude of the *R*^2^ of the direction GLM. The neurons were sorted by the preferred direction in the “go” time window. The color map on the right demonstrates the full saturation color for that preferred direction in the “go” time window. *R*^2^ = 0.69 was the largest *R*^2^ for the direction GLM, and was used to define the full saturation color. ***C***, Pearson correlation of direction tuning curves at one time versus another, among the 274 neurons in *A* and *B*. Colors indicate the mean correlation across these neurons. White contour indicates the area with where correlation was significantly larger than zero with Bonferroni correction (*p* < 0.05).

Despite our ability to decode both the vertical and horizontal direction during planning (Fig. 6*B*), single neurons in the FOF were not tuned equally to all movement directions (Fig. 8*A*). Firstly, there were significantly more neurons that preferred horizontal directions than those who preferred vertical directions (*χ*^2^(1, *N* = 274) = 76.91, *p* = 0 for the go window). Interestingly, rats mostly made errors into the same left/right side as instructed (Fig. 1*G*), which indicates that the over-representation of horizontal directions might be behaviorally relevant. Consistent with previous findings, there was no significant difference between the numbers of neurons preferring the ipsilateral and the contralateral side (*χ*^2^(1, *N* = 274) = 0.88, *p* = 0.34, Erlich et al., 2011). However, when we consider only the vertical aspect of tuning, there were significantly more neurons whose vertical preference was downwards rather than upwards (*χ*^2^(1, *N* = 274) = 5.37, *p* = 0.02 for the go window). The direction tuning of single neurons were stable across time. The *R*^2^s of the direction GLM remains high over hundreds of milliseconds (Fig. 8*B*), and the Pearson correlation of direction tuning curves were significantly larger than zero spanning over the fixation and the movement period (Fig. 8*B*).

**Figure 9.**
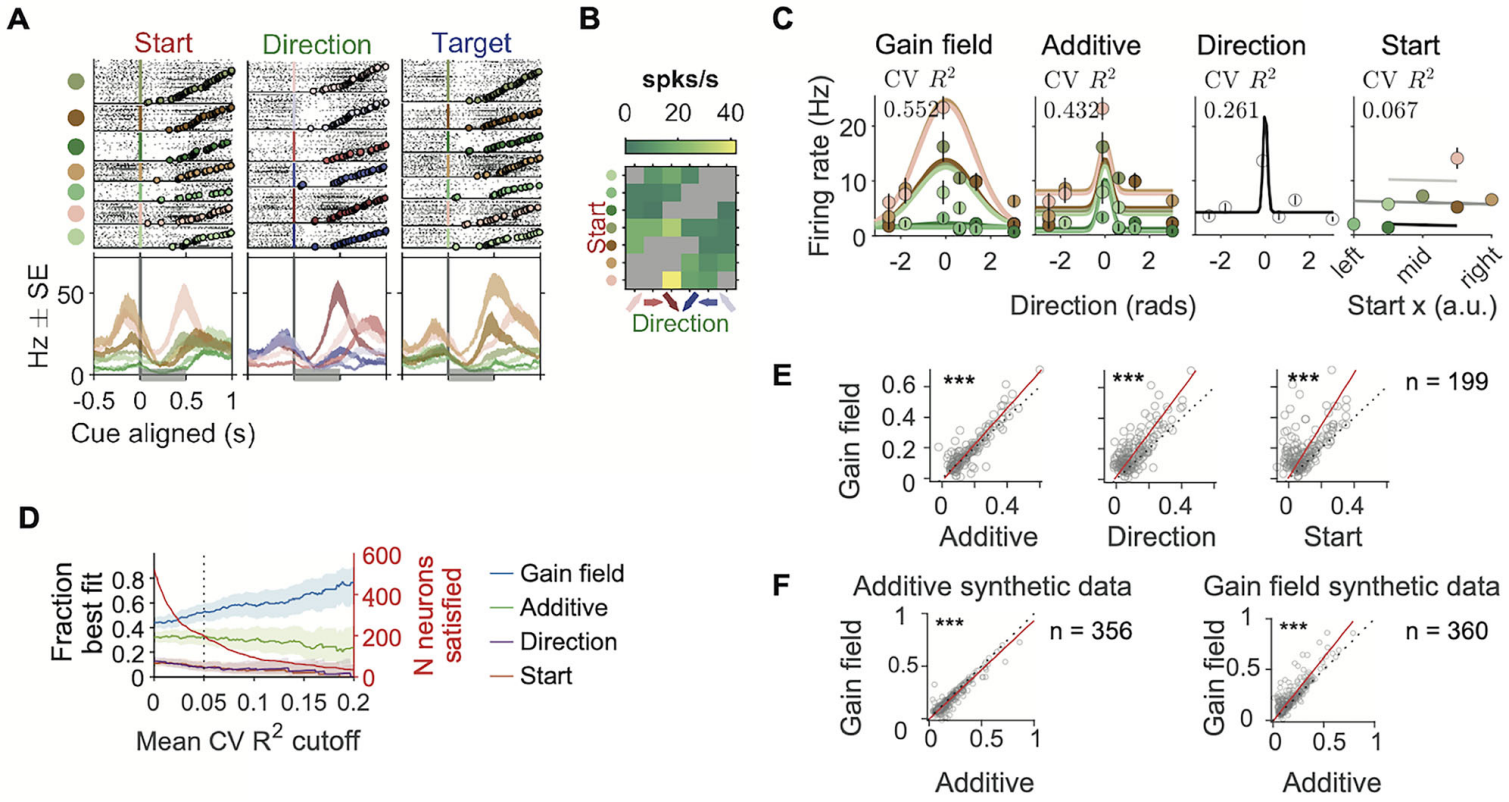
The spatial selectivity of FOF neurons were best explained by the gain-field model. ***A***, Raster plots and PETHs of an example neuron. Trials were grouped by the start position (left panel), the direction (middle panel), and the target position (right panel). The gray bar at the bottom indicates the 500 ms time window after the visual cue onset, the time window used for firing rate estimation, and model fitting in *B–E*. ***B***, Estimated mean firing rate in each movement trajectory using a maximum a posterior estimator, same as in Figure 3. ***C***, Predicted firing rates of four fit models (lines) and the mean and s.e. (circles and error bars) of firing rates in each trial condition. *CV R*^2^, cross-validated *R*^2^ (see Methods for definition). ***D***, Left axis, fraction of neurons best fit by a specific model, among neurons whose mean cross-validated *R*^2^s over the four models was larger than the *x*-axis indicated value. Best fit was defined as having the largest cross-validated *R*^2^ among the four models. Error bars were 95% confidence intervals of the binomial distribution. Right axis, neurons that crossed the mean *CV R*^2^ criteria for each *x*-axis value. ***E***, Each panel plots the cross-validated *R*^2^s of the *x*-axis model versus the *y*-axis model. Each circle indicates a neuron. The red line indicates the total least-square fit to the data. The dashed black line marks the diagonal. Only neurons whose mean of cross-validated *R*^2^s in the four models were larger than 0.05 were included (*n* = 199). The mean *R*^2^ in the gain-field model was larger than the *x*-axis models in all of these panels. ***, *p* < 0.001 (gain field vs additive, *p* = 8 × 10^−5^; gain field vs direction, *p* = 2 × 10^−5^; gain field vs start, *p* = 2 × 10^−5^. Permutation test against the null hypothesis that the Fisher-z transformed cross-validated *R*^2^s in the *x*-axis model and the *y*-axis model was not significantly different from zero, with 10^5^ shuffles). ***F***, The cross-validated *R*^2^s of the additive model versus the gain-field model in synthetic neurons, designed to have additive or gain-field selectivity, respectively. Only synthetic neurons whose cross-validated *R*^2^s in the four models were larger than 0.05 were included. Both panels had *p* = 2 × 10^−5^ (permutation test same as in E).

**Figure 10.**
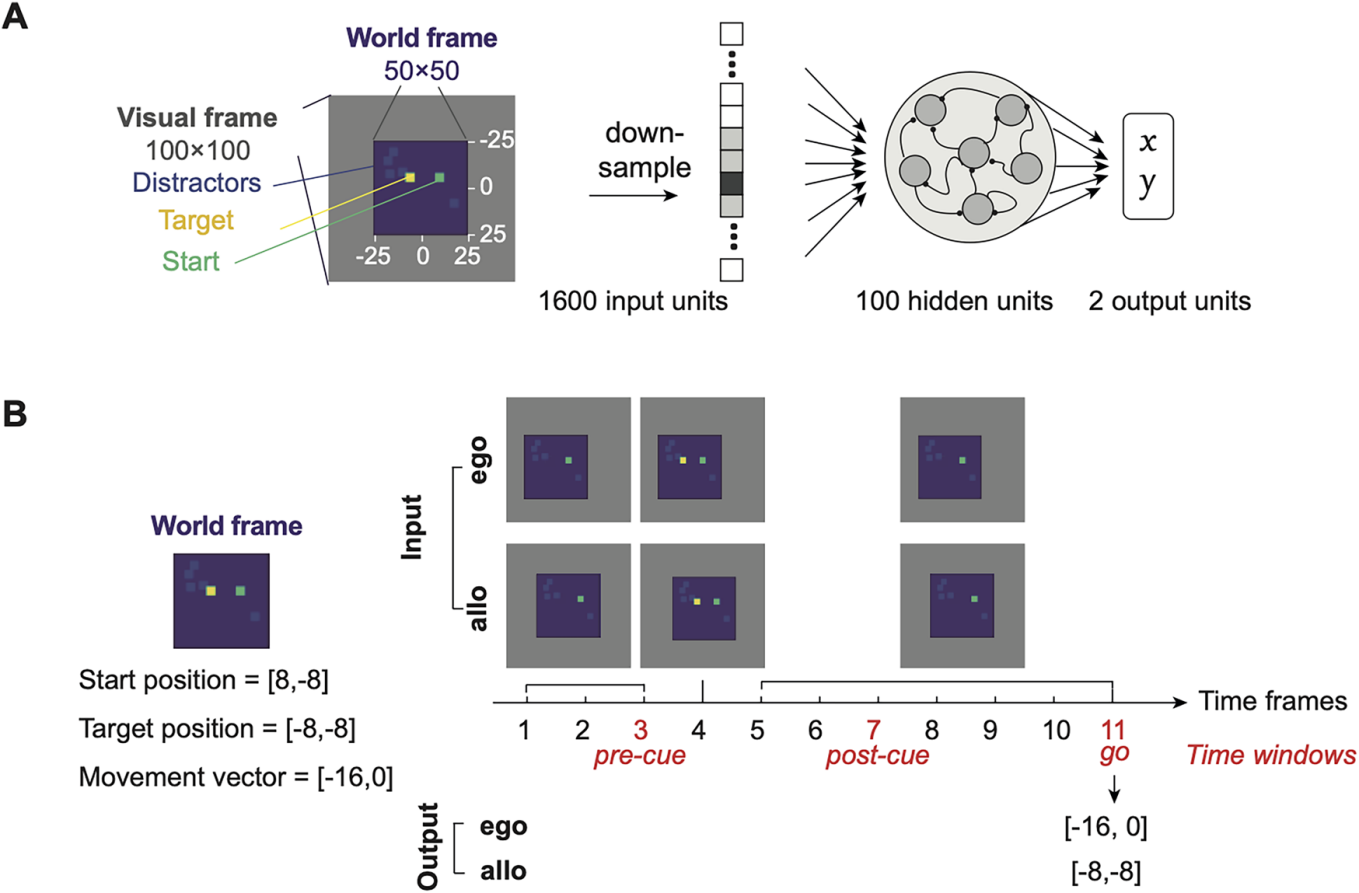
Design of RNN simulations with four types of input–output contingencies. ***A***, The schematics of the models. The input to the RNN was a figure that was down-sampled and flattened. The elements in the figure was intensity-coded and pseudo-colored for demonstration purpose. The output was a two-element vector of the *x* and *y* coordinates. ***B***, The timeline of a trial and the input–output contingency for an example world frame. The trial consists of 11 time frames, where the target port was transiently visible on the 4th frame. In ego-input models, the start position was always at the center of the visual frame, whereas in allo-input models, the world frame was always at the center of the visual frame. On time frame 11, allo-output models were required to output the target position and ego-output models were required to output the movement vector. The time frames 3, 7, and 11 were designated as the pre-cue, post-cue, and go windows to compare with data from FOF neurons.

**Figure 11.**
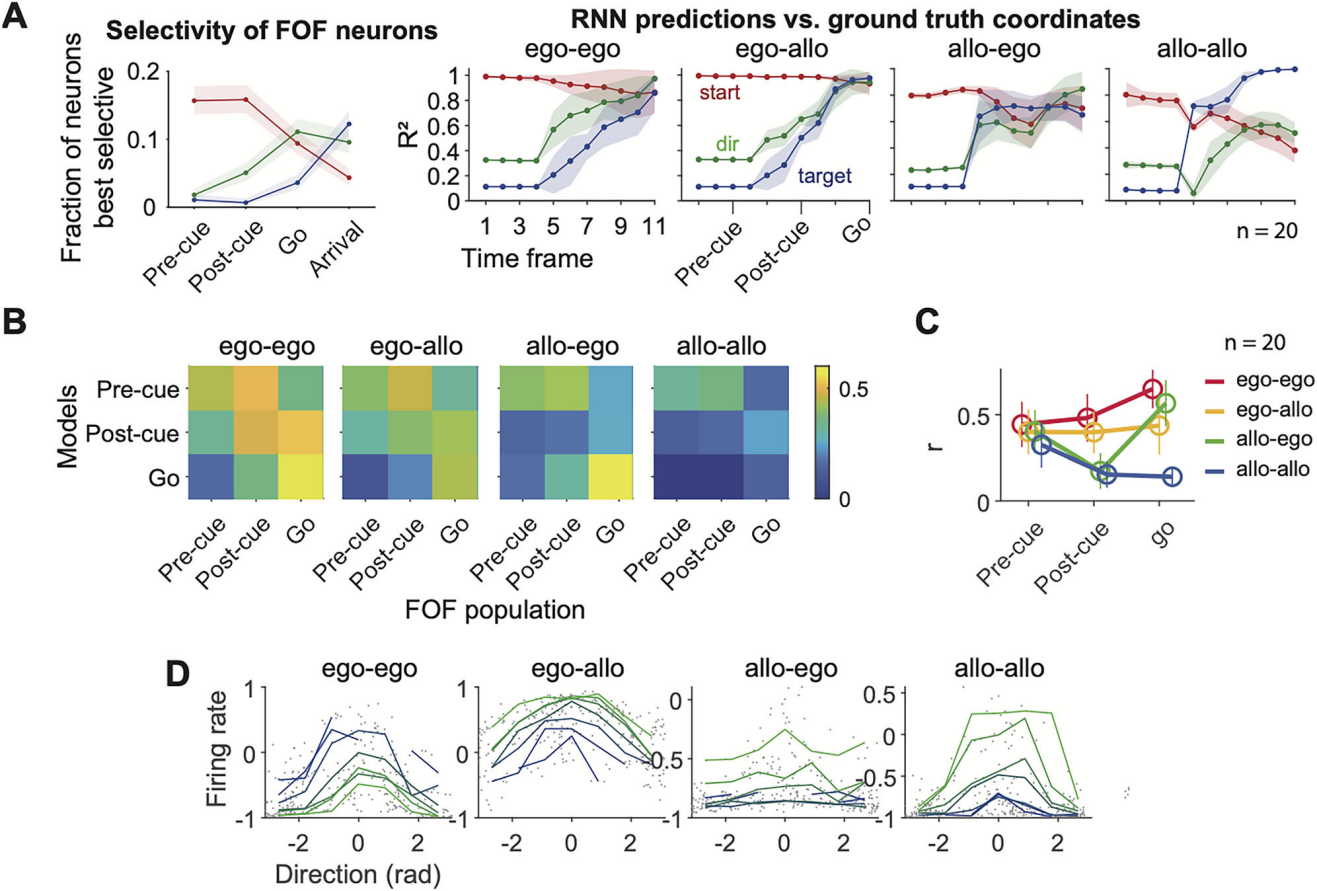
FOF population activity was most similar to a recurrent network with self-centered input and self-centered output. ***A***, Left, same as Figure 4*A*. Right, the cross-validated *R*^2^s from a linear model that decodes the start position, movement vector, or target position from hidden unit activities. Shaded area denoted the standard error of the mean over 20 training and testing epochs. The ego-ego network had the most similar temporal pattern of decoding accuracy as the FOF neurons. ***B***, The representational similarity between the FOF neural population activity and the population activity in RNNs, computed with the first four principle components of pseudopopulation activity in the FOF neural data and the network hidden unit data. Colors denote the representation similarity averaged across 20 RNN training epochs. ***C***, The representation similarity between the FOF neural population activity and the network hidden unit activity at the diagonal of *B*. Circles and error bars were mean and s.d. from 20 training and testing epochs. Representation similarity of the ego-ego models were significantly higher than other models in post-cue and go windows (Table 3). ***D***, Example units in the four networks that had gain-field-like selectivity to start position and direction. Each dot is a trial and each line is the average over trials with the same start position which is indicated by color.

### Mixed selectivity of movement direction and head position

In the previous analyses, we examined the encoding and decoding of one spatial variable at a time, although single FOF neurons seemed to encode the conjunction of multiple spatial variables by visual inspection (Fig. 3). Nonlinear mixed selectivity supports flexible readout by allowing high-dimensional representation of information from multiple sources (Rigotti et al., 2013). For example, a nonlinear transformation is required to compute distance ($\sqrt{x^{2}+y^{2}}$) from neurons that encode *x* and *y* position.

Gain-field models are a common function form of nonlinear mixed coding of spatial information. They are so named since the information of one source multiplicatively modulates the information of another source (Andersen and Mountcastle, 1983; Andersen et al., 1985, 1990; Salinas and Sejnowski, 2001). For example, the primate FEF encodes a gain-field mixture of the initial eye-in-orbit position and the saccade vector (Cassanello and Ferrera, 2007, 2010). Following this, we hypothesized that FOF activity might be described as a gain-field mixture of the current position and the upcoming movement direction. We tested the hypothesis by comparing four encoding models: a pure direction tuning model (“Direction” model, Eq. 1, see Methods), a pure position tuning model (“Start” model, Eq. 2), an additive mixed tuning model (“Additive” model, Eq. 4), and a gain-field mixed tuning model (“Gain-field” model, Eq. 3).

For each neuron, we fit spike counts in the 0–500 ms time window aligned to the visual cue onset to the four function forms (Fig. 9*A–C*), and quantified the goodness of fit with cross-validated *R*^2^ (*CV R*^2^). The vast majority of neurons were categorized as best fit by the gain-field model, and the fraction of neurons best fit by a gain-field model grew when the comparison was restricted to more spatially selective neurons (Fig. 9*D*). Among neurons whose average *CV R*^2^s for the four models were larger than 0.05 (*n* = 199), the mean *CV R*^2^ for the gain-field model was significantly larger than all the other models (Fig. 9*E*, Table 2). The result was qualitatively the same for spike counts in the −300 to 500 ms window aligned to the go sound, as well as in the 500 ms window with maximum cross-trial-type variance aligned to the visual cue onset, demonstrating consistency across time. Thus, the majority of FOF neurons had nonlinear mixed selectivity to the self-centered and the world-centered spatial variables, consistent with findings in the primate frontal and parietal cortices during motor planning (Andersen and Mountcastle, 1983; Andersen et al., 1985, 1990; Salinas and Sejnowski, 2001).

**Table 2.** Difference of the Fisher-transformed *CV R*^2^s between model pairs for neurons whose mean *CV R*^2^ > 0.05 for all four models

*M*_1_	*M*_2_	*p* value	atanh(Model 1) − atanh(Model 2) Mean, [95% CI]
Gain field	Additive	8 × 10^−5^	0.0129, [0.0073,0.0228]
Gain field	Direction	2 × 10^−5^	0.0617, [0.0544,0.0776]
Gain field	Start	2 × 10^−5^	0.0753, [0.0649,0.0959]
Additive	Direction	2 × 10^−5^	0.0488, [0.0430,0.0588]
Additive	Start	2 × 10^−5^	0.0624, [0.0530,0.0777]
Direction	Start	2 × 10^−2^	0.0136, [0.0024,0.0259]

Permutation test for the null hypothesis that atanh(Model 1) − atanh(Model 2) = 0 with 10^5^ permutations.

**Table 3.** Comparing the representational similarity for the ego-ego model versus the other three models

	Pre-cue	Post-cue	Go
Ego-ego versus ego-allo	*t*(38.00) = 1.05	*t*(37.34) = 2.02	*t*(32.95) = 4.69
	*p* = 0.150	*p* = 0.025	*p* = 2.29 × 10^−5^
Ego-ego versu allo-ego	*t*(37.90) = 1.02	*t*(35.03) = 7.96	*t*(36.79) = 2.11
	*p* = 0.156	*p* = 1.14 × 10^−9^	*p* = 0.021
Ego-ego versus allo-allo	*t*(38.00) = 2.86	*t*(29.77) = 9.19	*t*(26.86) = 18.41
	*p* = 0.003	*p* = 1.68 × 10^−10^	*p* = 4.65 × 10^−17^

Welch’s *t*-test for the alternative hypothesis that the mean of the representational similarity between the ego-ego model and real neurons were higher than that between another model and real neurons.

### FOF population activity is most similar to a recurrent network with self-centered input and self-centered output

Gain fields have been suggested as a powerful computational mechanism for reference frame transformation, as it allows a downstream population to read out an arbitrary combination of the spatial information from different sources (Salinas and Abbott, 1995; Pouget and Sejnowski, 1997). That said, our data does not provide strong evidence whether the FOF is involved in the reference frame transformation from a self-centered target direction to world-centered target position or alternatively inherits target position representations from upstream regions. We turned to a computational modeling approach to gain insight into this question.

We analyzed the activity of four types of networks (*n* = 20 per class) trained on a task similar to the visually guided delayed-orienting task. The four types each had different input and output reference frame configurations: self-centered input and self-centered output (ego-ego); self-centered input and world-centered output (ego-allo); world-centered input and self-centered output (allo-ego); world-centered input and world-centered output (allo-allo). The inputs to the networks were a flattened image with four major elements: the visual frame (outer frame), the world frame (inner frame), the start port and the target port (Fig. 10*A*). There were 11 time frames in a trial. The target port was transiently visible at the fourth time frame, and the other three elements were visible throughout the 11 time frames. In ego-input networks, the start port was always at the center of the visual frame, whereas in allo-input networks, the world frame was always at the center of the visual frame (Fig. 10*B*). On time frame 11, ego-output models were required to report the movement vector coordinates, and allo-output models were required to report the target position coordinates in the world frame.

Ego to allo coordinate transformations were required in ego-allo and allo-ego models but not in the ego-ego and allo-allo models. This was because in the ego-ego and allo-allo cases, the final output coordinates were both simply the position of the target port in the visual frame. Thus in these scenarios, there was a one-to-one mapping between the final output and the the position of the target port in the flattened image input. However, in the ego-allo and allo-ego cases, there were no such one-to-one mappings. These models needed to calculate the difference between two dynamic elements in the flattened image input. The ego-allo model needed to calculate the difference between the target port and the origin of the world frame, and the allo-ego model needed to calculate the difference between the start port and the target port. That said, all models required the networks to learn to extract specific visual information (in a pixels coordinate system) and output the information in a Cartesian coordinate system.

We decoded the start position, target position, and the movement vector from the hidden unit activity during test trials with a linear decoder, similar to what we did for real neurons. Visually, we found that the movement vector could be decoded earlier than the target position from the ego-ego and the ego-allo networks, similar to the temporal profiles in the FOF population. The temporal order was not clear in the allo-ego model and was reversed in the allo-allo model (Fig. 11*A*).

To quantify the similarity between recorded neurons and the four models, we applied RSA between the FOF population activity patterns and hidden unit activity patterns (Fig. 11*B,C*). RSA is a powerful technique to compare activity in networks (real or artificial) with different architecture, as long as the same stimuli can be presented to each network (Kriegeskorte, 2008). The idea behind RSA is to first estimate correlation between stimulus evoked activity patterns in each network and then to compare the patterns of correlations across the two networks. The ego-ego model activity was significantly more similar to the real neural population than the other three models (*p* < 0.0001 in at least one time window; Table 3). The key features driving this effect are that direction information emerges early (in the post-cue period) and is stronger than target position at the time of the go cue. Only the ego-ego model has both of these features. This supports our findings that planning in the FOF takes place in egocentric coordinates. It further hints that the allocentric positional information found in the FOF may not be central to its role in this task.

Interestingly, all four models showed gain-field-like activity in some of the hidden units (Fig. 11*D*), indicating that gain-field modulation is not uniquely associated with reference frame transformation that require vector subtraction within the network. It suggests that in a visual scene with multiple features (the visual vs world frame, start and target pixels and distractors), even identifying a specific element and transforming its position into a Cartesian coordinate system is sufficient for gain fields to emerge. Thus, despite the observation of allocentric spatial information in the FOF during the movement period and gain modulation of egocentric tuning by position, our modeling results indicate that these features can emerge even in a network that is not performing an ego-allo (or allo-ego) coordinate transform.

## Discussion

Motor planning in rodents has previously been studied with tasks where the action space is either very high dimensional (such as navigating a maze or an open field) or very low dimensional (such as 2AFC or go-nogo tasks). We took an intermediate approach with an orienting task that involved six movement directions and seven head positions. We observed encoding of both egocentric and allocentric spatial parameters at the single-neuron and population level in the FOF. At the population level, two distinct 2D maps could be decoded from the neural activity in the FOF: a 2D map of current position on the poke wall and a 2D map of the future movement vector. The encoding of allocentric start position, egocentric movement direction and allocentric target position emerged sequentially over the trial. Despite the presence of allocentric information in the FOF, preparatory activity was in the egocentric reference frame.

This work presents a substantial advance in the conception of the function of the FOF. Firstly, we established that preparatory activity in the FOF is encoded in a self-centered (egocentric) reference frame, consistent with the consensus view of preparatory activity in the primate FEF (Schall, 2009). This preparatory activity is relatively abstract compared to the specific muscle sequence required for a movement: the upcoming direction of movement could be decoded ignoring the start position (Fig. 6*B*). This finding is again consistent with findings from the FEF: auditory and visually guided saccades produce similar activity in the FEF even though they result in different eye/head kinematics (Russo and Bruce, 1994). Thus, although the preparatory activity is self-centered, neither the activity in FEF nor FOF are likely to represent a motor command, but instead represents an abstract command to shift attention to a part of space. Alternatively, it might not represent a command at all, but represent a utility or priority map (Bisley and Mirpour, 2019) which is used to generate movement commands in a downstream region, like the superior colliculus.

Our result builds on a recent interesting finding of world-centered spatial context modulating self-centered movement planning in rat M2 during navigation (overlapping anatomically with the region we define as FOF; Olson et al., 2020). The authors deduced that the M2 integrates spatial information toward the updating of planned movements. However, their animals were not explicitly cued on each trial about the goal location or direction while navigating along a triple T-maze, which precluded them from demonstrating the temporal pattern of spatial encoding of different reference frames. With a good experimental control over planning versus execution phases in our task, we demonstrated that planning is done preferentially in egocentric coordinates. Moreover, our comparison between the dynamics of the FOF neurons and the artificial neural networks using RSA casts doubt on the idea that the FOF transforms spatial information from one reference frame to another. The second difference is that we use a vertically oriented port wall instead of maze, which allowed us to demonstrate that the FOF contains two 2-dimensional maps (azimuth and elevation): one for position and one for direction (Fig. 6*B*).

Although it may appear that the FOF encodes the current position in a place-cell like fashion, there are “allocentric” features of the box that could lead to this kind of tuning. For example, the right mystacial whiskers might touch the box edge when animals are in the right-most port or there could be visual cues that change (distance to the wall or box edges) depending on the position of the subject in the ports. That said, most of the position-tuned neurons had spatially smoothed tuning. For example, firing highest for the middle left port, but responding moderately to top left or bottom left (Fig. 3*K*). While it is not impossible for sensory responses to have this spatially smooth tuning (e.g., distance from an odor cue), it seems parsimonious to describe these as allocentric position-tuned responses. Further experiments are required to better understand the nature of the allocentric tuning observed.

Subjects made few errors in the task, but when they did, they mostly made up/down errors rather than errors to the left or right (Fig. 1*G*), and two of the four rats made significantly more errors downwards than upwards (Fig. 1*H*). At the neural level, there was an over-representation of horizontal relative to vertical direction coding in the FOF, and when considering the vertical tuning, an over-representation of downwards versus upwards directions (Fig. 8). Although we do not have evidence for a causal relationship between these behavioral and neural observations, one might speculate that the horizontal over-representation in the FOF might be behaviorally relevant. In the hippocampus and entorhinal cortex, representations of the trajectories taken by rats are over-represented, even when the trajectories fill 3D space (Hayman et al., 2011; Jovalekic et al., 2011; Grieves et al., 2020). In our task, all instructions had a horizontal component and overall the poke wall is wider than it is tall which could produce a horizontal bias, but it seems surprising that this would generate such an extreme bias in neural tuning. In contrast to our findings, there were more pitch (up/down) tuned neurons than azimuth (left/right) tuned neurons reported in the M2 in rats foraging in a large arena (Mimica et al., 2018, 2023). There were two key differences between our experiment and theirs: first, they recorded from a larger anterior-posterior range of M2; second, our rats were under tight experimental control in a small enclosed training box whereas their rats were foraging freely in an open arena. In nature, when rodents are foraging in the open, attending to overhead events is essential to avoid predation (Wallace et al., 2013). It would be interesting to record the same neural populations in two tasks: one like ours and one like theirs to see if neurons in M2 can dynamically shift their tuning based on task demands.

The directional asymmetries in neural encoding and behavior suggest that a radially symmetric bump attractor model is insufficient as a computation model for the task (Wimmer et al., 2014). Plausible models for movement direction planning might be multiple discrete attractor models or ring attractor models that are asymmetric in connectivity weights: they might have stronger connectivity from upper direction preferring neurons to lower direction preferring neurons than the reverse, and have stronger connectivity between neurons that prefer the same left/right side than those preferring different left/right sides.

We found that allocentric and egocentric information is multiplexed as gain fields at the level of individual neurons. Gain modulation is a nonlinear process in which neurons combine information from two or more sources. Gain fields have been observed in a plethora of primate cortical and subcortical brain regions (for review, see Salinas and Sejnowski, 2001), including the frontal and parietal eye fields during saccade planning (Andersen et al., 1985; Cassanello and Ferrera, 2007, 2010). While conjunctive coding of allo- and ego-centric information has been found in rodent para- and post-subiculum and the medial entorhinal cortices (Peyrache et al., 2017; Gofman et al., 2019), they were not explicitly modeled as gain fields even though theoretical work suggests that they might take that form (Bicanski and Burgess, 2020). Our observations of gain fields in the rat FOF was largely similar to those in the primate literature.

Gain-field modulation were suggested to support reference frame transformation (Zipser and Andersen, 1988; Salinas and Abbott, 1995; Pouget and Sejnowski, 1997). According to the theory of gain-field modulation, a neural population downstream of a population with gain-field encoding could easily compute an arbitrary combination of the two spatial variables (Zipser and Andersen, 1988; Salinas and Abbott, 1995; Pouget and Sejnowski, 1997; Alexander et al., 2023). However, our comparison of recurrent networks showed that a recurrent network model whose input and output were both in the egocentric reference frame had the most similar activity to the FOF neurons: including representing allocentric activity and gain fields (Fig. 11*D*). These results further support our conclusion that planning in the FOF takes place in an egocentric coordinate frame, although the multiplexed spatial encoding may support spatial information integration in downstream brain areas or online error correction which might be required for planning complex movements sequences.

Our task was inspired by paradigms widely used in non-human primates to study the neural mechanism of saccadic eye movements (Bruce and Goldberg, 1985) and rodent head orienting is, like a saccade a shift in overt attention, in that it redirects the sensory fields of vision, audition, olfaction and whiskers (McCluskey and Cullen, 2007; Monteon et al., 2010; Bush et al., 2016). Based on similarities in connectivity and function, the rodent FOF is a proposed functional analog to the primate FEF (Reep et al., 1987, 1990; Erlich et al., 2011; Ebbesen et al., 2018), although a strict correspondence between rodent and primate frontal regions may not exist (Wise, 2008; Barthas and Kwan, 2017). Our findings of gain field modulation of movement direction by initial head position support the analogy, as this is similar to the observation that eye-in-orbit position gain modulates the planned saccade vector in FEF (Cassanello and Ferrera, 2007, 2010). One may argue that for the FEF both the initial gaze direction (or eye-in-orbit position) and the saccade vector are egocentric, and most of the reports about spatial encoding in the FEF was egocentric (but see Bharmauria et al., 2020). However, body positions in primate experiments are typically restricted by a primate chair. Recent work in freely moving monkeys found widespread world-centered coding across frontal and prefrontal cortex (Maisson et al., 2022), including supplementary motor area and dorsolateral prefrontal cortex. Although FEF was not one of the regions they recorded from, the finding suggests that allocentric representations are common across the brain, when animals’ movements are not restricted. Thus, we might speculate that FEF saccade planning activity in monkeys freely moving in an environment could be modulated by the current position of subject in the environment.

The observations of gain fields in the FOF, as well as the sequential encoding of movement direction and target position, were largely consistent with observations in primate motor planning more generally (Andersen et al., 1985; Zipser and Andersen, 1988; Salinas and Sejnowski, 2001; Wang et al., 2007; Caruso et al., 2018; Bharmauria et al., 2020). However, despite decades of research, a full understanding of phenomena like gain fields and how they could causally contribute to reference frame shifts has been hampered by a lack of tools for precise perturbations for circuit dissection in primates (as discussed in Shenoy et al., 2013). In contrast, the neural circuits underlying basic phenomena of motor planning (i.e., spatial memory and movement initiation in 2AFC tasks), have been revealed in rodents models (Kopec et al., 2015; Li et al., 2016; Guo et al., 2017; Inagaki et al., 2019; Bharmauria et al., 2020; Duan et al., 2021; Yang, 2022). Our behavioral paradigm and neurophysiological observations provide a basis for employing the latest tools of rodent systems neuroscience to understand complex and ethological motor planning.

## Code Accessibility

Please visit https://github.com/erlichlab/fof-visually-guided to access the code used for analyses and to generate figures. Links to the data are available from the github repository.
